# Tidal pumping facilitates dissimilatory nitrate reduction in intertidal marshes

**DOI:** 10.1038/srep21338

**Published:** 2016-02-17

**Authors:** Yanling Zheng, Lijun Hou, Min Liu, Zhanfei Liu, Xiaofei Li, Xianbiao Lin, Guoyu Yin, Juan Gao, Chendi Yu, Rong Wang, Xiaofen Jiang

**Affiliations:** 1 State Key Laboratory of Estuarine and Coastal Research, East China Normal University, Shanghai 200062, China; 2 College of Geographical Sciences, East China Normal University, Shanghai 200241, China; 3 The University of Texas at Austin Marine Science Institute, 750 Channel View Drive, Port Aransas, Texas 78373, USA

## Abstract

Intertidal marshes are alternately exposed and submerged due to periodic ebb and flood tides. The tidal cycle is important in controlling the biogeochemical processes of these ecosystems. Intertidal sediments are important hotspots of dissimilatory nitrate reduction and interacting nitrogen cycling microorganisms, but the effect of tides on dissimilatory nitrate reduction, including denitrification, anaerobic ammonium oxidation and dissimilatory nitrate reduction to ammonium, remains unexplored in these habitats. Here, we use isotope-tracing and molecular approaches simultaneously to show that both nitrate-reduction activities and associated functional bacterial abundances are enhanced at the sediment-tidal water interface and at the tide-induced groundwater fluctuating layer. This pattern suggests that tidal pumping may sustain dissimilatory nitrate reduction in intertidal zones. The tidal effect is supported further by nutrient profiles, fluctuations in nitrogen components over flood-ebb tidal cycles, and tidal simulation experiments. This study demonstrates the importance of tides in regulating the dynamics of dissimilatory nitrate-reducing pathways and thus provides new insights into the biogeochemical cycles of nitrogen and other elements in intertidal marshes.

The global nitrogen (N) cycle is affected by the production and widespread application of artificial N fertilizers^1^, of which approximately 60% is washed out of the soil into rivers or groundwater, primarily as nitrate (NO_3_^−^)^2^,^3^. The anthropogenic perturbation of the N cycle continues to cause serious environmental problems, ranging from eutrophication to hypoxia and stratospheric ozone loss^1^,^4^. Intertidal marshes are important conduits for the transfer of NO_3_^−^ from agricultural and other terrestrial sources into marine ecosystems because they are the transitional zones between land and sea^5^. NO_3_^−^ is dissimilated within intertidal sediments by microbial processes, including denitrification, anaerobic ammonium (NH_4_^+^) oxidation (anammox) and dissimilatory NO_3_^−^ reduction to NH_4_^+^ (DNRA). All are important NO_3_^−^ transformation pathways. Heterotrophic denitrification, which uses NO_3_^−^ to respire organic matter, produces N_2_ and, to a lesser extent, the greenhouse gas N_2_O^3^, whereas anammox, the anaerobic autotrophic oxidation of NH_4_^+^, uses nitrite (NO_2_^−^) or NO_3_^−^ as an electron acceptor to yield N_2_^6^,^7^. Denitrification and anammox remove NO_3_^−^ as gaseous products, thereby reducing the risk of eutrophication. In contrast, DNRA retains N within the ecosystem by recycling NO_3_^−^ into NH_4_^+^^8^. Thus, the balance of these dissimilatory NO_3_^−^-reduction processes, relating to geochemical and physical factors, has great biogeochemical consequences for N retention, plant growth and the climate^9^. However, the relative dominance of these NO_3_^−^-reduction pathways and the underlying sustaining mechanisms are not yet defined in intertidal marshes.

Intertidal marshes constitute major portions of meso- and macrotidal estuaries and cover approximately 40 million hectares worldwide^10^. These marshes provide many important ecosystem services^11^, including storm protection, carbon sequestration, nurseries, breeding grounds for wildlife, and most importantly the removal and transformation of land-derived nutrients. These effects on nutrient dynamics account for 66% of their service values^12^,^13^. Intertidal marshes are exposed and submerged alternately during tidal cycles, giving rise to rhythmic material fluxes between the sediment surface and tidal water. These physical forces regulate the transport and transformation of nutrients near the sediment-tidal water interface (SWI)^14^. Additionally, tidal dynamics induce periodic fluctuations of the groundwater table^15^, which in turn contributes to the pulsed input of groundwater-transported solutes and particles into deeper marsh sediment depths^16^,^17^. These processes, associated tightly with tidal cycles, are referred to here as “tidal pumping”.

We hypothesized that tidal pumping plays an important role in mediating the NO_3_^−^-reducing processes in intertidal zones via regulating the transportation of nutrients as well as electron donors and accepters, based on marsh hydrology^17^. The Yangtze Estuary was selected as our study area to test this hypothesis (Supplementary Fig. 1 and Fig. 1I); it is one of the largest estuaries in the world and contains extensive intertidal marshes. We (1) analyzed the vertical variations of potential denitrification, anammox and DNRA rates using ^15^N isotope-tracers, (2) examined the biodiversity and abundance of related functional bacterial groups in core sediments with molecular techniques, and (3) quantified nutrient profiles, groundwater fluctuations and N component dynamics over flood-ebb tidal cycles. A tidal simulation experiment was also conducted to assess the effects of tidal dynamics on intertidal NO_3_^−^-reducing processes. This study demonstrates the importance of tides in maintaining dissimilatory NO_3_^−^ reduction and provides novel insights into the biogeochemical cycles of N and other elements in intertidal marshes.

## Results

### Depth distributions of dissimilatory NO_3_^−^-reducing activities

**Figure 1:**
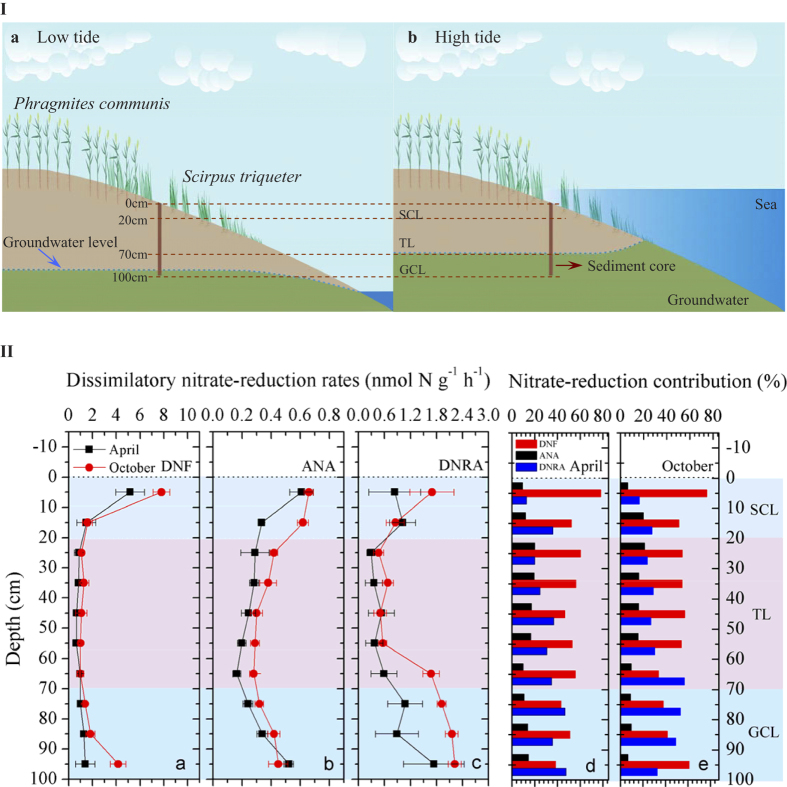
Vertical profiles of dissimilatory nitrate-reduction activities in intertidal marshes. (**I**) Schematic of the sampling cores at low tide (**a**) and high tide (**b**) in intertidal marshes. (**II**) The vertical distribution patterns of potential denitrification (DNF) rates (**a**), anammox (ANA) rates (**b**), DNRA rates (**c**), and their relative contributions to total nitrate reduction in April (**d**) and October (**e**), respectively. SWI: Sediment-water interface; SCL: SWI controlled layer in which sediment is primarily affected by overlying tidal water over tidal cycles; TL: Transition layer; GCL: Groundwater controlled layer in which sediment is primarily affected by groundwater fluctuation over tidal cycles. Sediment-layer identification (SCL, TL, and GCL) is based on the depth distributions of sediment water content (Supplementary Fig. 2). Error bars indicates s.d. (n = 3).

The potential rates of denitrification, anammox and DNRA were investigated in whole sediment cores (0–100 cm) based on sediment slurry incubation experiments in combination with ^15^N isotope-labeling techniques. The denitrification activity showed that the highest rates occurred in surface sediments (5.17 nmol N g^−1^ h^−1^ in April and 7.80 nmol N g^−1^ h^−1^ in October) (Fig. 1IIa). Below the top layer, denitrification decreased dramatically to the lowest values at 50–60 cm (0.64 nmol N g^−1^ h^−1^) in April and at 60–70 cm (0.99 nmol N g^−1^ h^−1^) in October. Notably, denitrification activity was stimulated again at a deeper layer and increased to 1.40 nmol N g^−1^ h^−1^ (April) and 4.16 nmol N g^−1^ h^−1^ (October) at a sediment depth of 100 cm. The potential activity patterns for anammox resembled those for denitrification. The highest potential anammox rates occurred in the surface layer (0.61 nmol N g^−1^ h^−1^ in April and 0.66 nmol N g^−1^ h^−1^ in October) (Fig. 1IIb). Anammox decreased with sediment depth to the lowest values at 60–70 cm (0.16 nmol N g^−1^ h^−1^ in April and 0.28 nmol N g^−1^ h^−1^ in October) and then rebounded with depth and reached 0.52 nmol N g^−1^ h^−1^ (April) and 0.45 nmol N g^−1^ h^−1^ (October) at 100 cm. DNRA decreased from the surface (0.84 nmol N g^−1^ h^−1^ in April and 1.69 nmol N g^−1^ h^−1^ in October) down to a sediment depth of 50–60 cm (0.37 nmol N g^−1^ h^−1^ in April and 0.56 nmol N g^−1^ h^−1^ in October; Fig. 1IIc). Below 70 cm, DNRA activity was stimulated to a greater degree than denitrification and anammox, and increased to 1.74 nmol N g^−1^ h^−1^ (April) and 2.22 nmol N g^−1^ h^−1^ (October) at the deepest layer sampled in the sediment cores. Thus, dissimilatory NO_3_^−^-reduction processes were all enhanced with depth, an observation not reported previously in intertidal systems. We speculated that this increase in NO_3_^−^-reducing activities in deep core zones might be stimulated by allochthonous substrates transported by groundwater, a factor that varies in response to tidal dynamics.

**Figure 2:**
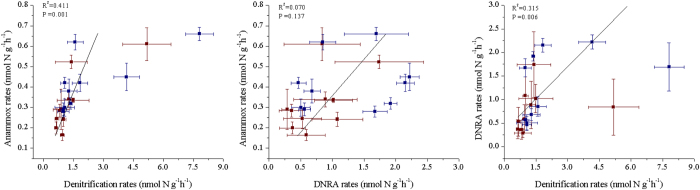
Pearson’s correlations among potential denitrification, anammox, and DNRA rates in intertidal marshes. Blue: April; Wine: October. Error bars indicates s.d. (n = 3).

The estimated contribution of denitrification to total NO_3_^−^ loss (the sum of denitrification, anammox and DNRA) in the whole sediment cores varied from 38.2% to 78.1% in April and from 33.7% to 76.8% in October (Fig. 1IId,e). While significant, NO_3_^−^ removal by anammox was low relative to denitrification. It contributed 9.2–19.9% and 6.5–21.4% of total NO_3_^−^ loss in April and October, respectively. DNRA retained 12.7–47.5% (April) and 16.7–56.8% (October) of the reduced NO_3_^−^ in the systems by converting it to NH_4_^+^. Despite internal competition and overlapping resource needs, denitrification was correlated positively with anammox (*R*^*2*^ = 0.411, *P* = 0.001) and DNRA (*R*^*2*^ = 0.315, *P* = 0.006), whereas the latter two pathways were not correlated significantly with each other (*R*^*2*^ = 0.070, *P* = 0.137; Fig. 2).

### Vertical distributions of NO_3_^−^-reducing microbial communities

**Figure 3:**
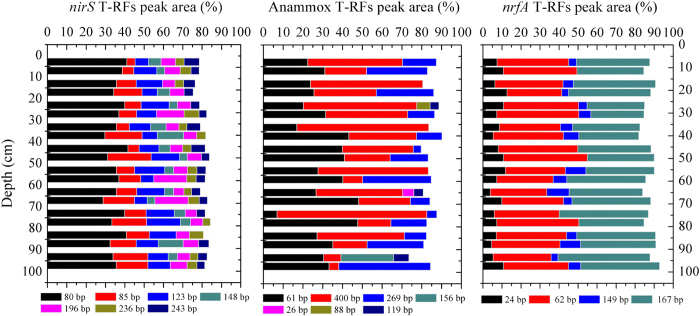
Community compositions and vertical distributions of denitrifiers (*nirS* gene), anammox bacteria (16 S rRNA gene) and DNRA bacteria (*nrfA* gene) in the intertidal sediments of the Yangtze Estuary based on the relative contributions of major T-RFs (Peak area > 4%) to total integrated area of peaks in the chromatograms. April sample: the first bar at each depth; October sample: the second bar at each depth.

Communities of dissimilatory NO_3_^−^ reducers in intertidal sediment cores were analyzed using PCR-based clone library and terminal restriction fragment length polymorphism (T-RFLP) analyses (Fig. 3 and Supplementary Table 1). The communities of *nirS* (cytochrome *cd1*-type NO_2_^−^ reductase)-harboring denitrifiers in the whole sediment cores were stable and dominated by T-RF of 80 bp (accounting for 28.8–41.7%), which were affiliated closely (87.4–98.0% similarities) with *nirS* clones retrieved from sediments of Chesapeake Bay (KC293444)^18^, Jiaozhou Bay (EU048464)^19^, Pearl River Estuary (HQ882339), and a constructed wetland (EF558459)^20^ (Fig. 3 and Supplementary Fig. 3). Two known anammox bacterial genera-*Candidatus Scalindua* (*S. sorokinii*, and *S. wagneri*, with 93.9–100% similarities) and *Candidatus Kuenenia* (*K. stuttgartiensis*, with 93.7–100% similarities) were detected (Supplementary Fig. 4). The anammox community in the sediment cores remained stable, with *Scalindua* dominating in most samples (50.2–83.5%; Fig. 3). The only exception was observed at 90–100 cm, where *Scalindua* and a novel genus (accounting for 39.1% and 26.7%, respectively) co-dominated in April, whereas *Scalindua* and *Kuenenia* (accounting for 38.1% and 46.4%, respectively) co-dominated in October (Fig. 3). The novel genus observed in the deepest layer in April was outside the “anammox bacterial cluster” with only 77.5–80.5% similarities, and had no close representatives among cultivated organisms (Supplementary Fig. 4); thus, the possibility that this genus represents a yet-uncultivated anammox microbe cannot be excluded. Molecular analyses of the *nrfA* gene (encoding cytochrome C NO_2_^−^ reductase, commonly found in DNRA bacteria) showed that DNRA communities were co-dominated by T-RFs of 62 bp and 167 bp, which in combination accounted for 67.5–81.0% in the whole sediment cores (Fig. 3). Sequences with T-RF 62 bp were related distantly to *nrfA* clones from Colne estuarine sediments (AM408261, with 80.2% similarity), whereas no close relatives (<75% identity) of T-RF 167 bp were found in the current GenBank database (Supplementary Fig. 5). Again, no significant vertical variations in DNRA biodiversity were observed, suggesting that the community structure of NO_3_^−^ reducers in intertidal sediments was not influenced by tidal dynamics.

### Vertical distributions of NO_3_^−^-reducing bacterial abundance

**Figure 4:**
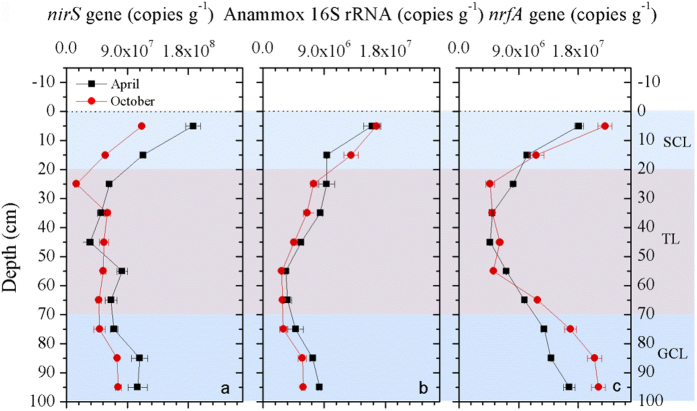
Bacterial gene abundances associated with dissimilatory nitrate-reduction processes in intertidal sediment cores. (**a**) Vertical distributions of denitrifier *nirS* gene abundance; (**b**) Vertical distributions of anammox bacterial 16 S rRNA gene abundance; (**c**) Vertical distributions of DNRA bacteria *nrfA* gene abudance. SCL: Sediment-water interface Controlled Layer; TL: Transition Layer; GCL: Groundwater Controlled Layer. Error bars indicates s.d. (n = 3).

Abundances of dissimilatory NO_3_^−^ reducers in intertidal sediment cores were analyzed using quantitative PCR (qPCR) techniques. The results showed that the abundance of *nirS*-harboring denitrifiers in intertidal sediment cores had a depth distribution resembling that of the potential denitrification rates (*R*^*2*^ = 0.698, *P* = 0.000; Supplementary Fig. 6). The highest abundance of the *nirS* gene occurred at the surface sediments (1.87 × 10^8^ in April and 1.11 × 10^8^ copies g^−1^ in October), which were 1.7–5.4 times higher than those detected at 20–70 cm depths (one-way ANOVA, *P* < 0.01; Fig. 4). Below 70 cm, *nirS* gene abundance increased again and reached 1.05 × 10^8^ (April) and 7.63 × 10^7^ (October) copies g^−1^ at the 100 cm sediment depth. Anammox bacterial 16S rRNA gene abundance was correlated positively with anammox potential rates (*R*^*2*^ = 0.356, *P* = 0.003; Supplementary Fig. 6). The near-surface values (1.60 × 10^7^ in April and 1.67 × 10^7^ copies g^−1^ in October) were 1.3–6.1 times higher than those observed in 20–70 cm layers (one-way ANOVA, *P* < 0.01). However, the population size of anammox bacteria increased below 70 cm to 8.30 × 10^6^ (April) and 5.91 × 10^6^ (October) copies g^−1^ in the deepest layer, as observed in the process rates (Fig. 4). Compared with denitrifiers and anammox bacteria, the abundance of DNRA bacteria was enhanced most intensively at deeper (below 70 cm) sediment depths where *nrfA* gene abundance was significantly higher than that observed in 30–60 cm layers (one-way ANOVA, *P* < 0.01). Additionally, significant correlations between *nrfA* gene abundance and DNRA rates were observed in the whole sediment cores (*R*^*2*^ = 0.251, *P* = 0.014; Supplementary Fig. 6). Thus, the overlap of the vertical distribution patterns of dissimilatory NO_3_^−^-reduction activities and associated bacterial abundances suggests that the elevated activity and population size at the surface and at depth may have resulted from tidal pumping, which can stimulate NO_3_^−^-reducing processes by transporting NO_3_^−^ and electron donors.

### Tidal effects on intertidal NO_3_^−^-reducing processes

**Figure 5:**
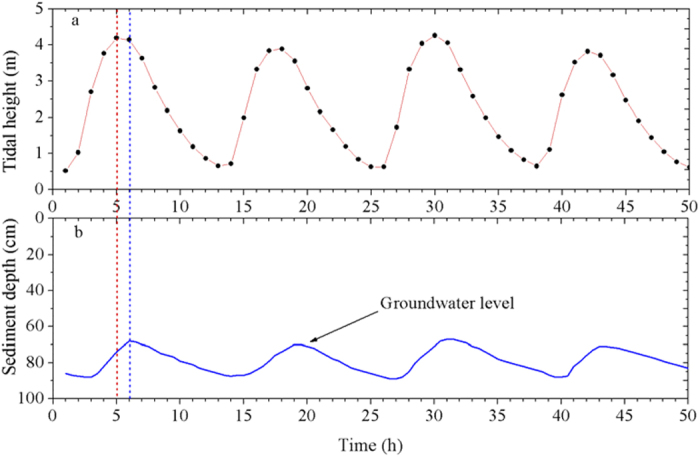
Tidal height (**a**) and associated groundwater fluctuations (**b**) over flood-ebb tidal cycles at the study area. The tidal heights are relative to the theoretical depth datum, which derived from the tide table for the study site; the data on the groundwater level were obtained from the *in situ* measurement from 8:00 AM on October 19 to 9:00 AM on October 21, 2013. Dotted lines show the time difference between groundwater-level fluctuation and tidal-height change.

**Figure 6:**
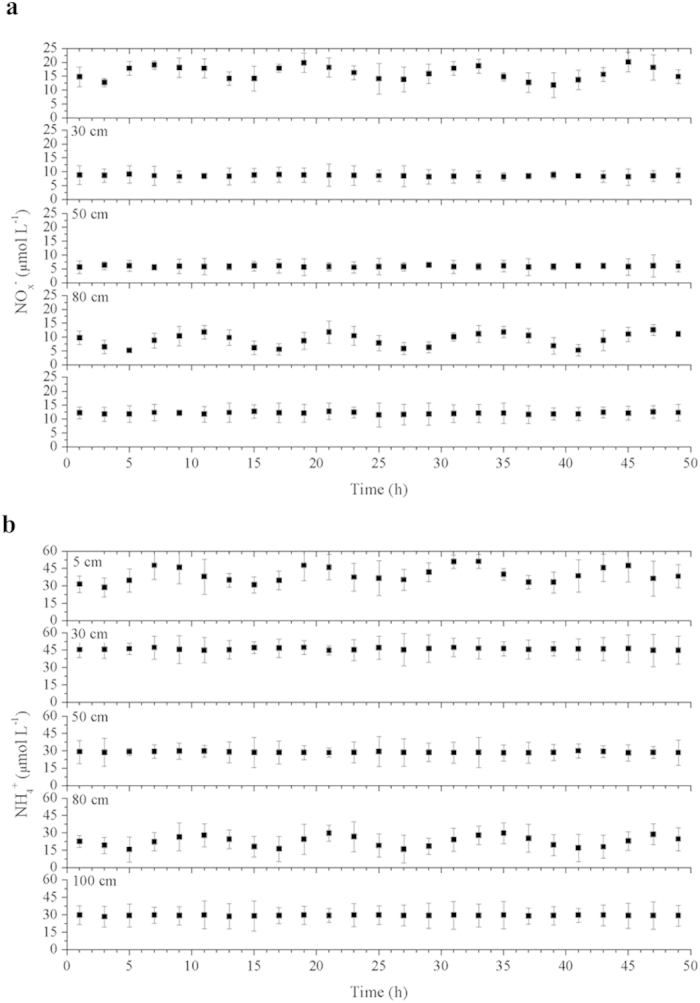
Concentrations of dissolved inorganic nitrogen ((**a**) NO_x_^−^ = NO_3_^−^ + NO_2_^−^; (**b**) NH_4_^+^) in different depth porewater over the 50 h tidal period. Triplicate samples were analyzed to get mean and standard deviation.

To obtain evidence of the tidal pumping effects on NO_3_^−^-reduction processes in intertidal marshes, groundwater levels were monitored over a 50 h spring-tide period. Groundwater oscillations were detected between sediment depths of approximately 70 cm and 90 cm, where NO_3_^−^-reducing activities and associated functional bacterial abundance were stimulated as the tide rose and fell over a tidal range of approximately 0.4–4.6 m (Fig. 5). Time-series curves showed that the groundwater-level fluctuations were markedly asymmetrical compared with the tidal-height changes but were characterized by a time lag of approximately 1 h, perhaps resulting from the differences between low and high tide swash conditions^21^. The concentrations of NH_4_^+^ and NO_x_^−^ (including NO_3_^−^ and NO_2_^−^) in porewater were also measured at depths of 5, 30, 50, 80, and 100 cm over the 50 h tidal period (Fig. 6). Significant temporal changes in NH_4_^+^ and NO_x_^−^ occurred at 5 cm and 80 cm depths (one-way ANOVA, *P* < 0.05). The observed NH_4_^+^ and NO_x_^−^ fluctuations in the SWI and groundwater oscillation layers over the tidal cycles imply that tidal pumping may provide substrates for NO_3_^−^ reducers in intertidal marshes.

**Figure 7:**
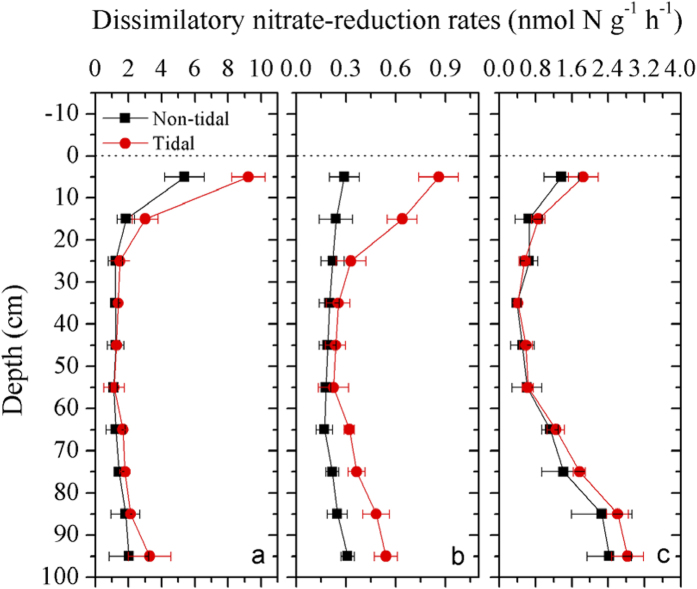
Vertical distributions of denitrification rates (**a**), anammox rates (**b**), and DNRA rates (**c**) in the tidal simulation experiment. Triplicate samples were analyzed to get mean and standard deviation. Tidal treatment was incubated in dark for 30 days under periodic immersion and emersion. Non-tidal was the control group without tidal treatment during the 30-day incubation.

The tidal effects on dissimilatory NO_3_^−^-reduction processes were assessed in a tidal simulation experiment (Fig. 7). Potential rates of denitrification in the tidal treatment ranged from 1.13 to 9.23 nmol N g^−1^ h^−1^, with a depth-integrated mean rate of 2.64 nmol N g^−1^ h^−1^. In contrast, the denitrification activity varied between 1.11 and 5.38 nmol N g^−1^ h^−1^ in the non-tidal (control) treatment, with a depth-integrated mean rate of 1.85 nmol N g^−1^ h^−1^. During the simulation experiment, the depth-integrated rate of denitrification in the non-tidal treatment decreased by approximately 29.8% compared with the tidal treatment. Potential anammox activity ranged from 0.23 to 0.86 nmol N g^−1^ h^−1^ and from 0.17 to 0.31 nmol N g^−1^ h^−1^ in the tidal and non-tidal treatments, respectively. During the simulation experiment, the depth-integrated rate of anammox in the non-tidal treatment (0.23 nmol N g^−1^ h^−1^) decreased by approximately 45.2% compared to the tidal treatment (0.42 nmol N g^−1^ h^−1^). Additionally, the depth-integrated contribution of anammox to total N_2_ production was relatively higher in the tidal (15.8%) than in the non-tidal (12.4%) treatments. DNRA activity ranged from 0.41 to 2.83 nmol N g^−1^ h^−1^ and from 0.39 to 2.43 nmol N g^−1^ h^−1^ in the tidal and non-tidal treatments, respectively. During the simulation experiment, the depth-integrated rate of DNRA in the non-tidal treatment (1.14 nmol N g^−1^ h^−1^) decreased by approximately 14.5% compared to the tidal treatment (1.34 nmol N g^−1^ h^−1^). Overall, the depth-integrated rate of total NO_3_^−^ reduction in the non-tidal treatment decreased by approximately 26.8% relative to the tidal treatment.

## Discussion

Dissimilatory NO_3_^−^-reduction pathways, including denitrification, anammox and DNRA, influence the recycling and removal of fixed N in aquatic ecosystems^3^,^6^,^8^. Denitrification was the dominant transformation pathway of NO_3_^−^, contributing approximately one half (53%, of the mean depth-integrated contribution of both seasons) to total NO_3_^−^ loss. Anammox played a significant but relatively minor role in NO_3_^−^ reduction, accounting for approximately 14% of total NO_3_^−^ loss. Anammox also contributed 20% of total N_2_ formation as compared to 80% supplied by denitrification. These data indicate that anammox is significant but not the dominant pathway for removing reactive N from intertidal marshes^22^−^25^. Potential DNRA accounted for 33% of total NO_3_^−^ reduction on average, implying that a large proportion of N was recycled internally and retained within the system. The potential rates of denitrification, anammox and DNRA and their estimated contributions to total NO_3_^−^ reduction in the study area were within the range of values observed in other tidal and estuarine ecosystems (Supplementary Table 2).

Dissimilatory NO_3_^−^-reduction activities were enhanced at both SWI and tide-induced groundwater fluctuating layers, as indicated by the isotope-tracing experiments (Fig. 1II). This pattern, not reported previously, suggests that tidal pumping may help sustain NO_3_^−^ reduction in intertidal zones. The intertidal sediment surface is an interface between a reducing sediment environment and oxidized zones in tidal water or the atmosphere. Therefore, it is an active zone for N transformations^26^−^28^. NO_3_^−^ reduction at the SWI controlled layer could be amplified directly by the diffusive input of NO_3_^−^ from overlying tidal water during inundation^28^ and/or indirectly by coupled nitrification/NO_3_^−^-reduction due to moderate O_2_ penetration from the marsh surface^17^. Beneath the SWI, NO_3_^−^-reducing rates decreased in response to the reduced availability of substrates. However, tide-induced groundwater fluctuation may cause a cyclical input of allochthonous reactive N to the GCL and thus stimulate NO_3_^−^ reduction^17^,^27^. These assumptions are supported by the vertical profiles of NO_x_^−^ and NH_4_^+^ in the present study (Supplementary Fig. 7). It is worth noting that shallow coastal aquifers are typically micro-oxic^17^, whereas no dissolved O_2_ was detected in the porewater of the groundwater-influenced layer in this study. The increases in Fe(III), Mn(IV), and Eh shown below the 70 cm depth are consistent with the idea that the oxygen delivered by groundwater is consumed rapidly to oxidize reduced ions, such as Fe(II) and Mn(II). The idea that the groundwater-derived O_2_ may support nitrification was not evident in this study (Supplementary methods and Supplementary Fig. 8).

NO_3_^−^ reducers exhibited the lowest activity in the transition layer, where substrate is insufficient. Additionally, this layer is a zone of minimum Eh values and high concentrations of Fe(II) and Mn(II) (Supplementary Fig. 7), suggesting some sulfide production from sulfate reduction in the intermediate depths^17^. Many studies have reported that sulfide may decrease denitrification rates via inhibiting N-monoxide and nitrous-oxide reductases^29^,^30^. Additionally, sulfide may irreversibly inhibit anammox bacteria^31^. These factors provide additional evidence for the rapid decrease in denitrification and anammox in the transition layer. Generally, sulfide and Fe(II) serve as potential electron donors and increase DNRA rates in sediments^32^,^33^. However, DNRA activity dropped to its lowest value at intermediate depths, indicating that substrate (e.g., NO_3_^−^ and organic matter) shortage may limit activity in this layer. NO_3_^−^ reducers (*nirS*-harboring denitrifiers, anammox bacteria and DNRA bacteria) did not vary significantly with depth in the intertidal sediment cores (Fig. 3). The community structure vertical stability indicates that the NO_3_^−^ reducers may be indigenous and adapt well to the local ecological niche. The only exception was observed in the 90–100 cm sediment layer, where the community structure of the anammox bacteria changed (Fig. 3). This deep layer transition may have resulted from frequent or long-term groundwater intrusions. The anammox communities in this deep layer varied seasonally (*P* < 0.05), showing that the sediment environment was dynamic, perhaps due to frequent tidal actions. In contrast, denitrifiers and DNRA bacterial biodiversity did not vary statistically in this deep layer, but further study is needed to reveal the underlying mechanism.

Although biodiversity of the nitrate reducing community did not exhibit significant vertical variations, the abundances of associated genes (*nirS* gene, anammox bacterial 16S rRNA gene, and *nrfA* gene) generally showed a consistent overall depth-distribution pattern, as observed in these process rates, which were also stimulated at the SWI and groundwater fluctuating layers (Fig. 4). Thus, the distribution pattern of targeted nitrate reducing genes provides molecular evidence supporting the hypothesis that tidal pumping facilitates dissimilatory NO_3_^−^ reduction in intertidal marshes. Although the presence of functional bacteria does not indicate that these organisms are active *in situ*, functional bacterial abundances often reflect recent process activities^34^. This conclusion is supported by the significant correlations between dissimilatory NO_3_^−^-reducing rates and associated bacterial gene abundances (Supplementary Fig. 6).

The groundwater fluctuations during tidal cycles provide evidence for tidal pumping effects. The NO_x_^−^ and NH_4_^+^ in the porewater of both SWI and groundwater-fluctuating zones varied significantly as the tide rose and fell (one-way ANOVA, *P* < 0.05; Fig. 6). The NO_x_^−^ and NH_4_^+^ concentrations in surface sediment porewater were higher during tidal immersion than during tidal emersion, perhaps due to the transport of NO_3_^−^ from NO_3_^−^-enriched tidal water to sediments, part of which was converted rapidly to NH_4_^+^ via DNRA^35^. Additionally, NH_4_^+^ consumption in surface sediments via nitrification is enhanced during tidal emersion compared with tidal immersion^26^,^36^, and thus might also contribute to the tidal changes of NH_4_^+^ and NO_x_^−27^. When the groundwater level rose, the NO_x_^−^ and NH_4_^+^ concentrations were higher at the 80 cm depth, perhaps due to recharging by the rising groundwater. These explanations may also be supported by the relationships of NO_x_^−^ and NH_4_^+^ concentrations with the fluctuations in tidal height and groundwater level (Supplementary Figs 9 and 10).

The tidal simulation experiment demonstrated that tidal pumping can maintain dissimilatory NO_3_^−^-reduction activity (Fig. 7). During the simulation incubation, the depth-integrated activity of NO_3_^−^ reduction decreased by approximately 27% when tidal pumping did not maintain the supply of N substrate for NO_3_^−^ reducers. Compared with N_2_-production pathways, DNRA decreased least in the non-tidal (control) treatment when no external nutrients were provided. DNRA has a higher affinity for NO_3_^−^/NO_2_^−^ than denitrification and may be favored in NO_3_^−^-limited environments^30^,^37^, which is attributed to the requirement of five electrons to reduce NO_3_^−^ in denitrification versus the eight required for DNRA^3^. DNRA may therefore outcompete denitrification in NO_3_^−^-limited environments, where the microbial organisms gain more energy from DNRA than from denitrification. DNRA tends to exceed denitrification and becomes the dominant NO_3_^−^-reducing pathway in the groundwater-controlled layer, where N is relatively limited and primarily depends on external input. Particularly in the non-tidal treatment, DNRA contributed up to 52% of the total NO_3_^−^ reduction in deep core zones (Supplementary Fig. 11) and thus retained most of the N recycled in this deep layer. DNRA did not outcompete denitrification in the transition layer even though NO_3_^−^ was limited, which may be attributed to the simultaneous shortage of N and organic electron donors, which would not favor DNRA^37^.

Denitrification, anammox and DNRA microbial pathways all compete for NO_3_^−^/NO_2_^−^, while denitrification and DNRA also compete for organic carbon. However, different NO_3_^−^-reduction processes might also interact for intermediate substrates^7^,^38^,^39^, as it is suggested that anammox may depend metabolically on the NO_2_^−^ supply from incomplete denitrification^7^. This hypothesis is supported by the significantly positive linear relationship (*R*^*2*^ = 0.411, *P* = 0.001) between anammox and denitrification rates observed in the present study (Fig. 2). However, the correlation between anammox and DNRA rates in intertidal sediment cores was not significant (*R*^*2*^ = 0.070, *P* = 0.137), showing that although DNRA can also produce NO_2_^−^ as an intermediate product for anammox bacteria^40^, it did not make a significant contribution compared with denitrification. A significant relationship between denitrification and DNRA activities was detected in our data (*R*^*2*^ = 0.315, *P* = 0.006), indicating that these two main NO_3_^−^-reduction processes did not inhibit each other via competition, but were both stimulated by tidal pumping at the SWI and groundwater fluctuating layers. The relative supply of NO_3_^−^/NO_2_^−^ in natural ecosystems is often controlled by nitrification, a two-step process that can yield either compound as the end product^37^. No significant correlation between potential nitrification and DNRA rates (*R*^*2*^ = 0.053, *P* = 0.168) was detected in this study, but significant relationships occurred between nitrification potential and NO_3_^−^-removal activities (denitrification: *R*^*2*^ = 0.739, *P* = 0.000; anammox: *R*^*2*^ = 0.481, *P* = 0.000; Supplementary Fig. 12). This trend suggests that nitrification might serve as the internal NO_3_^−^/NO_2_^−^ provider for gas-producing NO_3_^−^ reducers^35^, particularly in the SWI controlled layer^17^.

In summary, our data suggest that tidal pumping plays an important role in maintaining and controlling dissimilatory NO_3_^−^-reduction processes in intertidal marshes. They suggest new insights about how dissimilatory N dynamic processes interact in an intertidal environment subjected to periodic tidal inundation and exposure. Due to the worldwide essential role of intertidal marshes, this tide-driving mechanism may be important to the global N cycling. Future studies should focus on (1) defining the exact mechanisms of dissimilatory NO_3_^−^ transformation more precisely in intertidal marshes where redox conditions are affected by groundwater flux and (2) the effects of tidal pumping on the production of nitrous oxide (a long-lived and powerful greenhouse gas), which is associated closely with these N-transformation processes.

## Methods

### Study area and field sampling

The study site was located in the eastern intertidal flat of Chongming Island in the Yangtze Estuary. This site is the largest and most developed intertidal flat in the Yangtze Estuary, with an area of approximately 230 km^2^. The tide at the study area is semidiurnal, with a typical tidal range between 0.4 m and 4.6 m relative to the theoretical depth datum. NO_3_^−^ concentrations in the overlying water of the study area ranged from 60 to 120 μM with an average concentration of approximately 80 μM, while NH_4_^+^ concentrations varied from 5 to 30 μM with an average concentration of approximately 10 μM^28^,^41^. The sediment of the study site is primarily composed of silt, with smaller amounts of clay and sand^36^. In addition, *Phragmites communis*, *Scirpus triqueter* and other marsh plants are distributed in patches within the intertidal flat.

Six sediment cores (7.2 cm diameter and 100 cm depth) were collected from a small unvegetated (to exclude the potential influences of the vegetation roots) intertidal area (approximately 5 m^2^) in April and October 2013 (Supplementary Fig. 1). During the October sampling, porewater samples for the analyses of NH_4_^+^ and NO_x_^−^ were also collected from different sediment depths (5, 30, 50, 80, and 100 cm) with clay pipes (porewater samplers) every 2 h within two daily tidal cycles (approximately 50 h). Briefly, the clay pipes were inserted carefully into the sediments (three clay pipes at each depth). Then, 10 mL of porewater was collected at each time point via each clay pipe under the negative pressure driven by a hand-held vacuum pump, filtered on site through 0.22-μm Millipore filters (Millipore, Bedford, USA) and poisoned with saturated HgCl_2_ solution. The fluctuation of the groundwater level was measured with a sinusoidal porewater pressure sensor (YL-GSG, Shanghai, China) in a PVC pipe (1.5 m deep) buried near the sampling site^17^. All samples were transported on ice to the laboratory within 2 h. In the laboratory, the sediment cores were sliced immediately into subsamples at 10-cm intervals under helium in a glovebox and portions of each same depth were mixed to provide one composite sample. For each subsample composite, one fraction was stored at 4 °C to measure N transformation rates and sediment physicochemical characteristics and the other fraction was preserved at −80 °C for DNA extraction and molecular microbial analysis. Porewater samples were stored at 4 °C for NH_4_^+^ and NO_x_^−^ analyses.

### Determination of environmental parameters

Sediment redox potential (Eh) was measured using an FJA-6 ORP Depolarization Automatic Analyzer (Chuan-Di, Nanjing, China). The oxygen concentration in sediments was determined after sediment cores were collected using an OXY Meter S/N 4164 with an oxygen needle sensor (Unisense, Aarhus, Denmark). Sediment particles were analyzed with a Beckman Coulter LS13320 laser granulometer (USA). Salinity was measured with a YSI Model 30 salinity meter, after sediments were mixed with CO_2_-free deionized water at a sediment/water volume ratio of 1:2.5^7^. Exchangeable NH_4_^+^ and NO_x_^−^ were extracted from fresh sediments with 2 M KCl and measured spectrophotometrically on a continuous-flow nutrient analyzer (SAN plus, *Skalar Analytical* B.V., the Netherlands) with detection limits of 0.5 μM for NH_4_^+^ and 0.1 μM for NO_x_^−7^. The sedimentary organic carbon and nitrogen contents were determined using a CHN elementary analyzer (VVarioELIII, Elementary, Germany) after removing carbonate by leaching with 0.1 M HCl^36^. Concentrations of Fe(II) and Fe(III) in sediments were quantified by extracting 0.5 g of fresh sediment with 30 mL of 0.5 M HCl, followed by colorimetric determination^42^. Concentrations of Mn(II) and Mn(IV) in sediments were estimated by colorimetric determination after extraction from 0.5 g of fresh sediment with 30 mL of 1 M ammonium acetate (NH_4_OAc)^43^. All physiochemical parameters of sediments were analyzed in triplicate (Supplementary Fig. 7).

### Measurement of potential anammox and denitrification rates

Slurry experiments were conducted to measure the potential activities of anammox and denitrifying bacteria with a N isotope-tracing method^23^,^24^. In brief, slurries were made with fresh sediments and helium-purged tidal water at a volume ratio of 1:5 and then transferred into 12 mL helium-flushed glass vials (Exetainer, Labco, High Wycombe, Buckinghamshire, UK). Subsequently, the slurries were pre-incubated for approximately 24 h to eliminate residual NO_3_^−^, NO_2_^−^, and O_2_ at *in situ* temperature. After pre-incubation, these vials were divided into three groups, which were spiked through the septum of each vial with helium-purged stock solutions of (1) ^15^NH_4_^+^ (^15^N at 99.6%), (2) ^15^NH_4_^+^ + ^14^NO_3_^−^, and (3) ^15^NO_3_^−^ (^15^N at 99%), respectively. The final concentrations of ^15^N in each vial were approximately 100 μM. The incubation of slurries was stopped by injecting 300 μL of 50% ZnCl_2_ solution after 8 h. The concentrations of ^29^N_2_ and ^30^N_2_ produced over the incubation period were measured by membrane inlet mass spectrometry (MIMS), as described in ref. 7. The rates (expressed on a dry sediment weight basis) of both anammox and denitrification and their respective contributions to total N_2_ production were calculated using the methods developed in refs 22 and 44.

### Measurement of potential DNRA rates

Similar to the measurements of anammox and denitrification, slurry experiments were conducted to measure potential rates of DNRA with a N isotope-tracing method, as described in ref. 45. Briefly, slurries were prepared with fresh sediments and helium-purged tidal water at a volume ratio of 1:5 and then transferred into 12 mL helium-flushed Labco Exetainer vials. Subsequently, the slurries were pre-incubated to eliminate residual NO_3_^−^, NO_2_^−^, and O_2_ at *in situ* temperature. After a 24 h pre-incubation, the slurry vials were spiked with ^15^NO_3_^−^ (final concentration ca. 100 μM), and one-half of the replicates were designated as initial samples and preserved with 300 μL of 50% ZnCl_2_ solution. The remaining slurries were shaken (200 rpm) and incubated for approximately 8 h at near *in situ* temperature. At the end of the incubation, the remaining replicates as final samples were preserved with ZnCl_2_ solution, as described for the initial samples. The concentrations of ^15^NH_4_^+^ produced via DNRA in the slurry incubations were analyzed by a new Oxidation/MIMS (“OX/MIMS”) method^45^. Potential DNRA rates were estimated from the changes in ^15^NH_4_^+^ concentrations during the incubations^45^.

### Tidal effect simulation

A simulation experiment was conducted according to the characteristics of tidal dynamics at the study site. Briefly, six additional sediment cores (7.2 cm diameter and 100 cm depth) were collected from an unvegetated area of the study site in February 2015. After collection, these cores were transported to the laboratory within 2 h. After return to the laboratory, one half of the cores were incubated immediately in the dark at room temperature (20 °C) to test the tidal pumping effects. Briefly, these three sediment cores were pre-incubated for 30 d under periodic immersion and emersion. During the immersion, these three cores (with an open bottom end) were inserted approximately 30 cm deep into helium-flushed anaerobic artificial seawater (salinity: 1.0%; NH_4_^+^: 30 μM; and NO_3_^−^: 10 μM, based on the detected values in 100 cm deep porewater)^46^, whereas the surface of the core sediments was immersed with aerobic artificial seawater (salinity: 10%; NH_4_^+^: 10 μM; and NO_3_^−^: 80 μM, which were the averaged *in situ* values from previous investigations). After 6 h of immersion incubation, the cores were air dried for another 6 h. During emersion, the bottom end of these cores was sealed with butyl-rubber stoppers, whereas the sediment-core surfaces were exposed to air. The length of immersion/emersion was based on the average immersion/emersion periods observed in the intertidal flats of the Yangtze Estuary. In contrast, the remaining cores without tidal incubation were controls. During the incubation, the bottom ends of these cores were sealed with butyl-rubber stoppers, whereas sediment core surfaces were exposed to air. After 30 d pre-incubation, all of the cores were sliced into subsamples at 10 cm intervals to measure the potential dissimilatory NO_3_^−^-reduction rates, as described above.

### DNA extraction and gene amplification

Total genomic DNA was extracted from 0.25 g homogenized sediment samples using Powersoil^TM^ DNA Isolation Kits (MOBIO, USA) following the manufacturer’s instructions. We used the primers cd3aF^47^ and R3cd^48^ to target *nirS*-harboring denitrifiers (Supplementary Table 3). For anammox bacteria, a nested PCR approach was established, consisting of an initial PCR amplification (Pla46f/1390r)^49^,^50^ of Planctomycetales 16S rRNA gene followed by a second PCR (Amx368f/Amx820r)^51^,^52^ targeting anammox bacteria. PCR amplification of *nrfA* gene from DNRA bacteria was carried out with primers NrfAF2aw^53^ and NrfAR1^54^. PCR mixtures and thermocycling conditions were given in Supplementary Table 3. Appropriately-sized fragments were separated by electrophoresis in 1% agarose gels and purified using Gel Advance-Gel Extraction system (Viogene, China). The purified fragments were cloned using the TOPO-TA cloning kit (Invitrogen, USA) in accordance with the manufacturer’s instructions. Clones were selected randomly for further analysis.

### Sequencing and phylogenetic analysis

Screened clones were sequenced using an ABI Prism genetic analyzer (Applied Biosystems, Canada). The qualified nucleic acid sequences displaying more than 97% (for *nirS* and anammox bacterial 16S rRNA sequences) or 98% (for *nrfA* sequence) identity were grouped into one operational taxonomic unit (OTU) using software Mothur (version 1.23.0) by the furthest neighbour approach^55^. Neighbour-joining phylogenetic tree was created using MEGA software (version 5.03)^56^. The relative confidence of the tree topologies was evaluated by performing 1000 bootstrap replicates^56^. Clone sequences obtained in this study have been deposited in GenBank under accession numbers: KT444009-KT444439 for *nirS* gene; KJ658526-KJ658628 and KM577189-KM577278 for anammox bacterial 16S rRNA gene; and KT423178-KT423373 for *nrfA* gene.

### Terminal restriction fragment length polymorphism (T-RFLP)

PCR amplifications for T-RFLP analyses of *nirS* gene, anammox bacterial 16S rRNA gene and *nrfA* gene sequences were as described above, with the forward primers (cd3aF for *nirS*, Amx368f for anammox bacterial 16S rRNA, and NrfAF2aw for *nrfA*) fluorescently labeled with 6-carboxyfluorescein (FAM) at the 5′ end. Restriction digestion with enzyme *Hha*I (for *nirS* gene), *Alu*I (for anammox bacterial 16S rRNA gene) and *Mse*I (for *nrfA* gene) of 50 ng purified PCR products was performed at 37 °C overnight^25^,^57^,^58^. Digested samples were run on an ABI 3130X genetic analyzer and analyzed with the GeneMapper program (Applied Biosystems, Foster City, CA). All samples were digested in duplicate with independent PCR reactions and enzyme digestion to conform repeatability of the fragment patterns. Only peaks composing more than 4% of the chromatogram area were considered for further analysis.

### Real-time quantitative PCR

Gene abundance was determined by real-time qPCR, performed on an ABI 7500 Sequence Detection System (Applied Biosystems, Canada) using the SYBR green method. Triplicate qPCR reactions were set up for each sample, and the primers and thermocycling conditions are listed in Supplementary Table 3. Primers for quantification of *nirS* and *nrfA* genes were the same as for their clone library constructions. Abundance of anammox bacterial 16S rRNA gene was estimated with primers Amx-808-F/Amx-1040-R^59^. Plasmids carrying the targeted gene fragments were extracted from *E. coli* hosts using a Plasmid Mini Preparation Kit (Tiangen, China). Standard curves were obtained using gradient dilutions of standard plasmids containing targeted genes with known copy numbers. The specificity of the qPCR amplification was determined by the melting curve and gel electrophoresis to lower the possibility of over-estimation. Negative controls without DNA template were included in each amplification reaction. The qPCR amplification efficiencies (91.1–97.1%) and other calibration curve parameters (e.g., *R*^*2*^) are provided in Supplementary Table 4. Gene abundance was calculated based on the constructed standard curve, and then converted into copies per gram of dry sediment, assuming the DNA extraction efficiency was 100%.

### Statistical analysis

The biodiversity indicators (Shannon-Wiener and Simpson) and species richness Chao1 estimator were calculated for the clone libraries with Mothur program (version 1.23.0)^55^. The coverage of clone libraries was estimated by the percentage of the observed number of OTUs divided by Chao1 estimator. The distances between clone libraries were determined by UniFrac test, and the P-value were corrected for multiple comparisons using the Bonferroni correction (P-value was multiplied by the number of pairwise comparisons performed)^60^.

## Additional Information

**How to cite this article**: Zheng, Y. *et al.* Tidal pumping facilitates dissimilatory nitrate reduction in intertidal marshes. *Sci. Rep.* **6**, 21338; doi: 10.1038/srep21338 (2016).

## Supplementary Material

Supplementary Information
