# Supplementary material for: Tidal pumping facilitates dissimilatory nitrate reduction in intertidal marshes
Source: Sci Rep. 2016 Feb 17;6:21338. doi: 10.1038/srep21338 (PMC4756672; doi:10.1038/srep21338)
Supplement: Supplementary Information [file srep21338-s1.pdf]

**Supplementary Information for**

# Tidal pumping facilitates dissimilatory nitrate reduction in intertidal marshes

Yanling Zheng, Lijun Hou*, Min Liu*, Zhanfei Liu, Xiaofei Li, Xianbiao Lin, Guoyu Yin, Juan Gao, Chendi Yu, Rong Wang & Xiaofen Jiang

*Corresponding authors: Lijun Hou and Min Liu

## 1. Supplementary Methods

**Measurement of potential nitrification rates.** Potential nitrification rates were estimated in triplicate based on the method described in ref 1. Briefly, 2.0 g of wet weight sediment was added to 100-mL erlenmeyer flasks containing 30 mL of artificial seawater with *in situ* salinity. $NH_4Cl$ (for ammonium) and $KH_2PO_4$ (for phosphate) were amended to the flasks with final concentrations of 300 μM and 60 μM, respectively[2]. The suspension was incubated in the dark at near in situ temperature with continuous shaking at 120 rpm. During the incubations, subsamples were harvested at 0, 4, 8, and 12 h, respectively. Subsequently, they were centrifuged, filtered and immediately frozen for the analysis of nitrate (plus nitrite). The potential nitrification rates (expressed on a dry sediment weight basis) were calculated based on the linear increase of nitrate concentrations in suspension during the incubations[1].

**Determination of bacterial and archaeal amoA gene abundance.** Bacterial and archaeal *amoA* gene abundance was determined using real-time quantitative PCR (qPCR), which were performed in triplicate on an ABI 7500 Sequence Detection System (Applied Biosystems, Canada) with the SYBR green qPCR method. The primer set composed of amoA-1F and amoA-2R was used for the amplification of the AOB *amoA* gene[3]. Arch-amoAF and Arch-amoAR were used to amplify the AOA *amoA* gene[4]. The primer sequences and thermocycling conditions used for amplification were listed in Supplementary Table 3. Plasmids carrying the targeted gene fragments were extracted from *E.coli* hosts using a Plasmid Mini Preparation Kit (Tiangen, China). Concentrations of plasmid DNA were measured using a Nanodrop-2000 Spectrophotometer (Thermo, USA). Standard curves were obtained using gradient dilutions of standard plasmids containing targeted genes with known copy numbers. The specificity of the qPCR amplification was determined by the melting curve and gel electrophoresis. The qPCR amplification efficiencies, as well as other calibration curve parameters (e.g., $R^2$) were given in Supplementary Table 4. Negative controls containing no template DNA were subjected at the same time to detect and exclude any possible contamination or carryover. *amoA* gene abundance was calculated based on the constructed standard curve, and then converted into copies per gram of dry sediment, assuming the DNA extraction efficiency was 100%.

## 2. Supplementary Figures and Tables

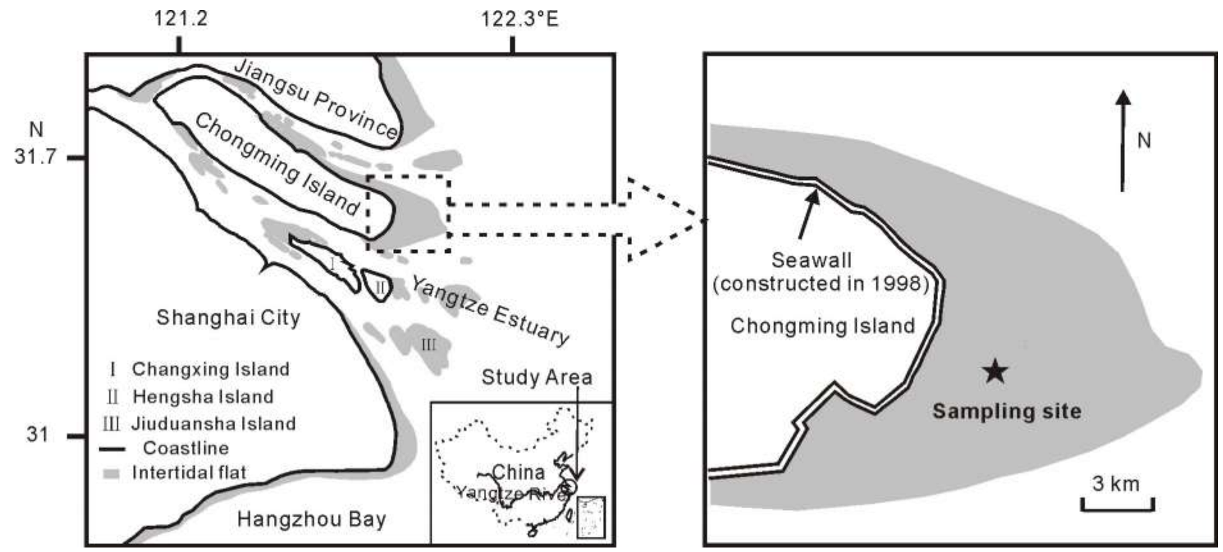


**Supplementary Figure 1** Study area. This figure shows the geographical location of the Yangtze Estuary in China and the sampling site. The map was created with ArcGIS 10.1.

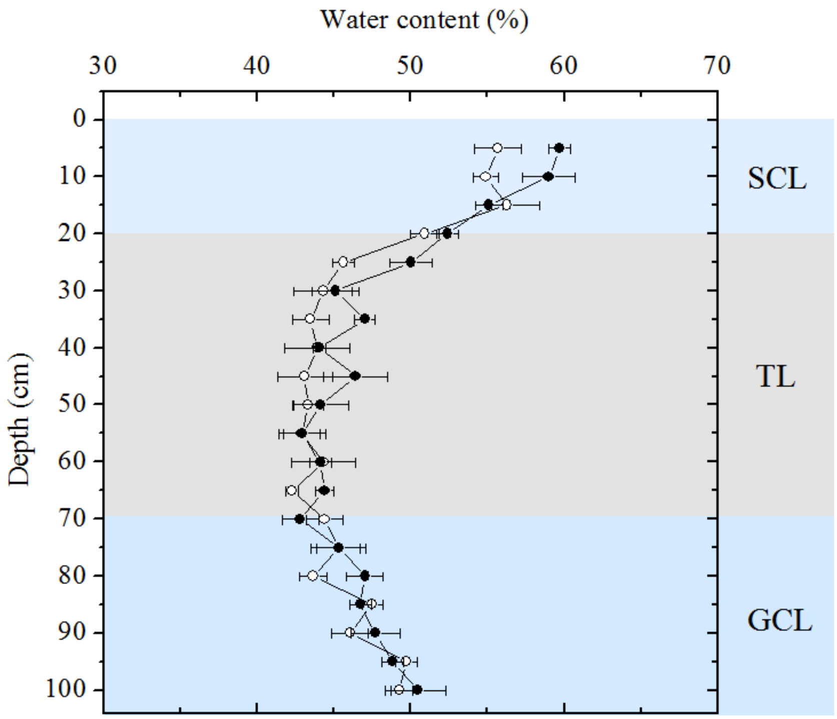


**Supplementary Figure 2** Vertical distributions of sediment water content in April (black circle) and October (open circle). This figure shows that the surface sediment (about 0-20 cm) near the sediment-water interface (SWI) is primarily affected by overlying tidal water over tidal cycles while the deeper sediment (about 70-100 cm) is primarily affected by groundwater fluctuation over tidal cycles. Therefore, based on the depth distributions of sediment water content, the sediment cores were divided into three layers: SWI controlled layer (SCL), Transition layer (TL), and Groundwater controlled layer (GCL). Error bars indicates s.d. (n = 3).

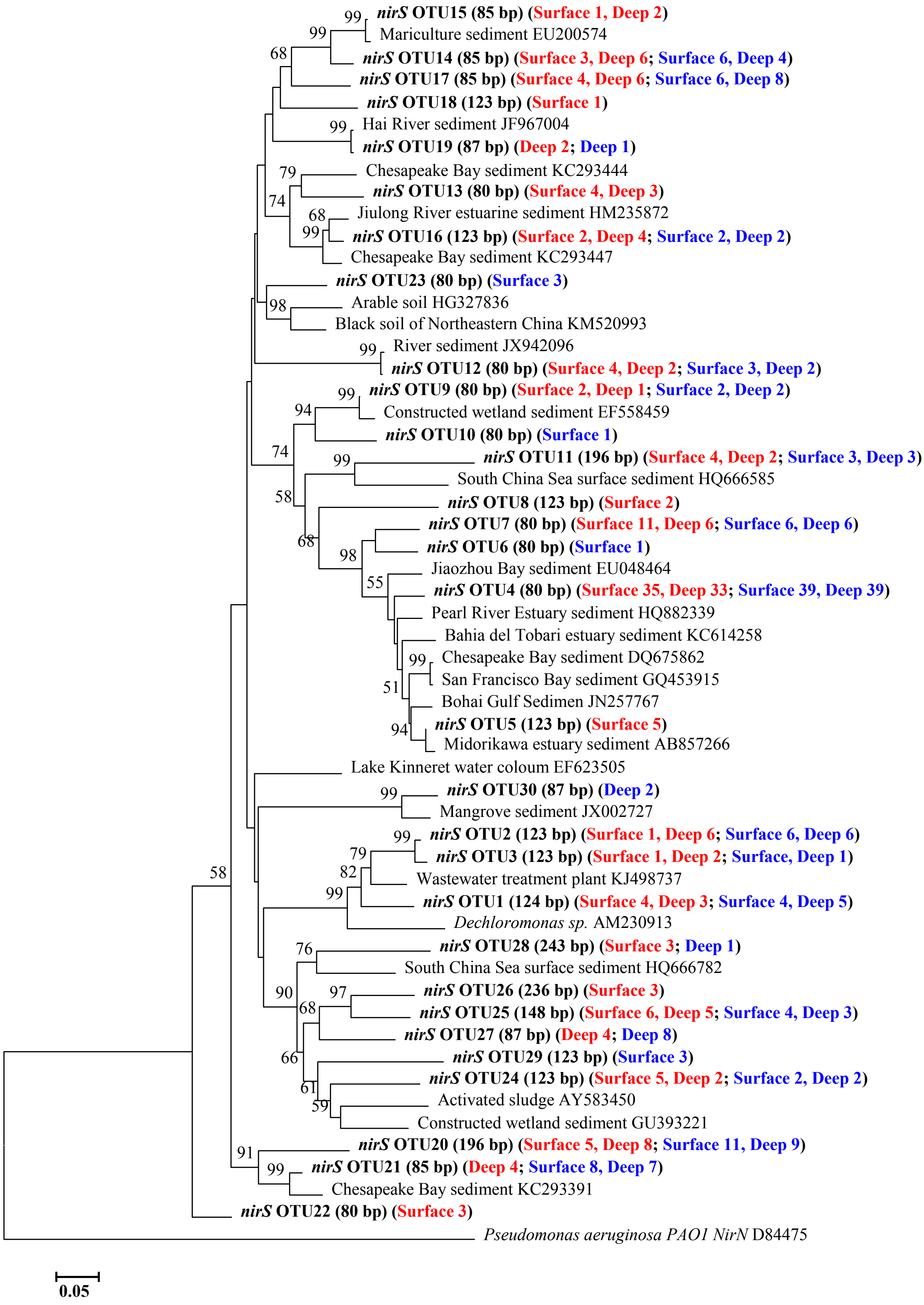

nirS OTU15 (85 bp) (Surface 1, Deep 2)
Mariculture sediment EU200574
nirS OTU14 (85 bp) (Surface 3, Deep 6; Surface 6, Deep 4)
nirS OTU17 (85 bp) (Surface 4, Deep 6; Surface 6, Deep 8)
nirS OTU18 (123 bp) (Surface 1)
Hai River sediment JF967004
nirS OTU19 (87 bp) (Deep 2; Deep 1)
Chesapeake Bay sediment KC293444
nirS OTU13 (80 bp) (Surface 4, Deep 3)
Jiulong River estuarine sediment HM235872
nirS OTU16 (123 bp) (Surface 2, Deep 4; Surface 2, Deep 2)
Chesapeake Bay sediment KC293447
nirS OTU23 (80 bp) (Surface 3)
Arable soil HG327836
Black soil of Northeastern China KM520993
River sediment JX942096
nirS OTU12 (80 bp) (Surface 4, Deep 2; Surface 3, Deep 2)
nirS OTU9 (80 bp) (Surface 2, Deep 1; Surface 2, Deep 2)
Constructed wetland sediment EF558459
nirS OTU10 (80 bp) (Surface 1)
nirS OTU11 (196 bp) (Surface 4, Deep 2; Surface 3, Deep 3)
South China Sea surface sediment HQ666585
nirS OTU8 (123 bp) (Surface 2)
nirS OTU7 (80 bp) (Surface 11, Deep 6; Surface 6, Deep 6)
nirS OTU6 (80 bp) (Surface 1)
Jiaozhou Bay sediment EU048464
nirS OTU4 (80 bp) (Surface 35, Deep 33; Surface 39, Deep 39)
Pearl River Estuary sediment HQ882339
Bahia del Tobari estuary sediment KC614258
Chesapeake Bay sediment DQ675862
San Francisco Bay sediment GQ453915
Bohai Gulf Sedimen JN257767
nirS OTU5 (123 bp) (Surface 5)
Midorikawa estuary sediment AB857266
Lake Kinneret water coloum EF623505
nirS OTU30 (87 bp) (Deep 2)
Mangrove sediment JX002727
nirS OTU2 (123 bp) (Surface 1, Deep 6; Surface 6, Deep 6)
nirS OTU3 (123 bp) (Surface 1, Deep 2; Surface, Deep 1)
Wastewater treatment plant KJ498737
nirS OTU1 (124 bp) (Surface 4, Deep 3; Surface 4, Deep 5)
Dechloromonas sp. AM230913
nirS OTU28 (243 bp) (Surface 3; Deep 1)
South China Sea surface sediment HQ666782
nirS OTU26 (236 bp) (Surface 3)
nirS OTU25 (148 bp) (Surface 6, Deep 5; Surface 4, Deep 3)
nirS OTU27 (87 bp) (Deep 4; Deep 8)
nirS OTU29 (123 bp) (Surface 3)
nirS OTU24 (123 bp) (Surface 5, Deep 2; Surface 2, Deep 2)
Activated sludge AY583450
Constructed wetland sediment GU393221
nirS OTU20 (196 bp) (Surface 5, Deep 8; Surface 11, Deep 9)
nirS OTU21 (85 bp) (Deep 4; Surface 8, Deep 7)
Chesapeake Bay sediment KC293391
nirS OTU22 (80 bp) (Surface 3)
Pseudomonas aeruginosa PAO1 NirN D84475
99
99
68
99
79
74
68
99
98
99
99
94
74
99
58
68
98
55
99
51
94
99
99
79
82
99
58
76
90
97
68
66
61
59
91
99
0.05

**Supplementary Figure 3** Neighbor-joining phylogenetic tree of denitrifier *nirS* gene sequences, with the *nirN* gene from *Pseudomonas aeruginosa PAO1* (accession no. D84475) used as an outgroup. Bootstrap values greater than 50% of 1000 resamplings are shown near nodes. The scale indicates the number of nucleotide substitutions per site. GenBank accession numbers are shown for sequences from other studies. Numbers in parentheses followed each OTU indicate the number of sequences recovered from surface and (or) deep part of the sediment cores in April (red) and October (blue). OTUs are defined by <3% divergence in nucleotides.

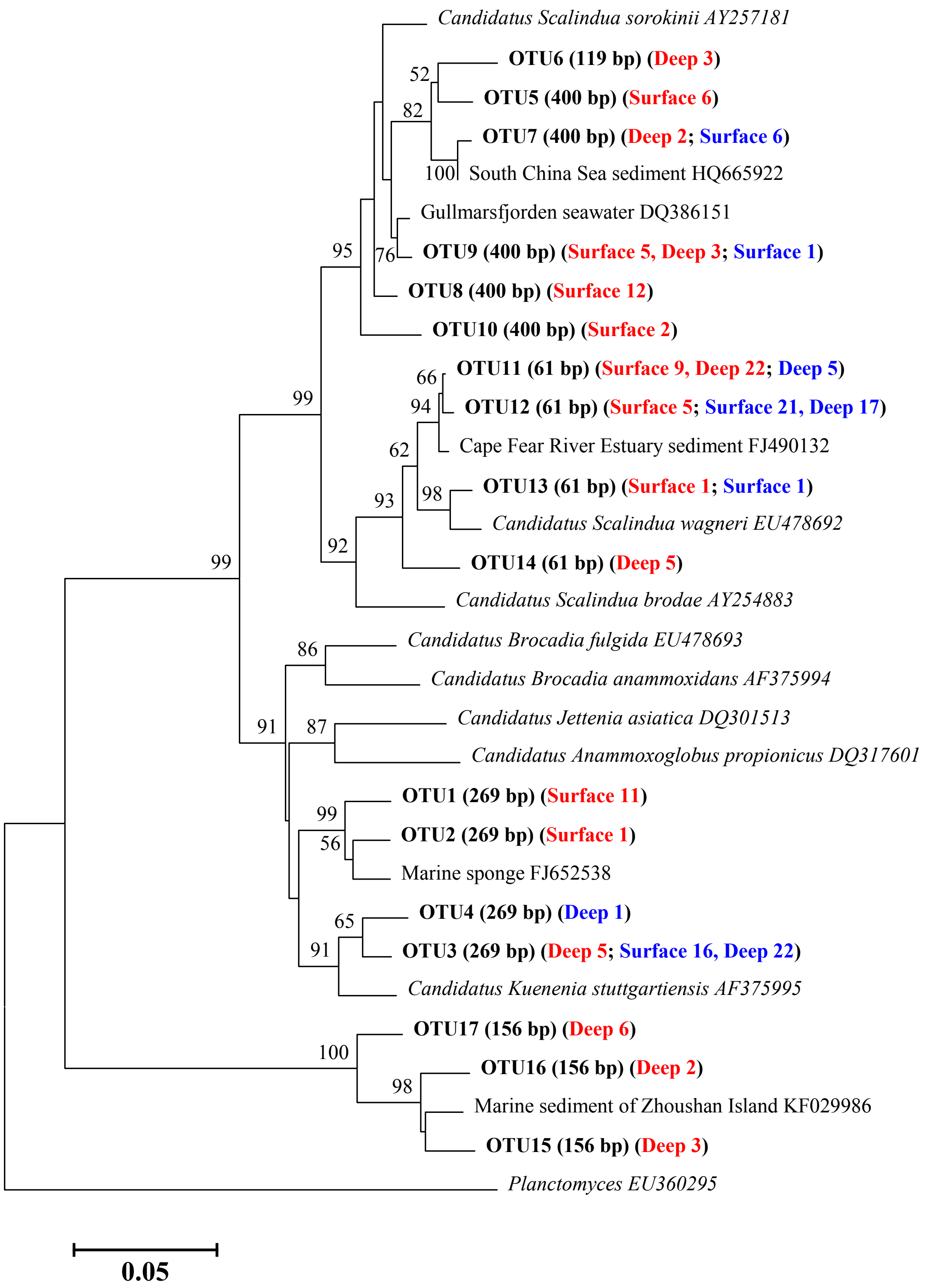


**Supplementary Figure 4** Neighbor-joining phylogenetic tree of anammox bacterial 16S rRNA gene sequences. Bootstrap values greater than 50% of 1000 resamplings are shown near nodes. The scale indicates the number of nucleotide substitutions per site. GenBank accession numbers are shown for sequences from other studies. Numbers in parentheses followed each OTU indicate the number of sequences recovered from surface and (or) deep part of the sediment cores in April (red) and October (blue). OTUs are defined by <3% divergence in nucleotides.

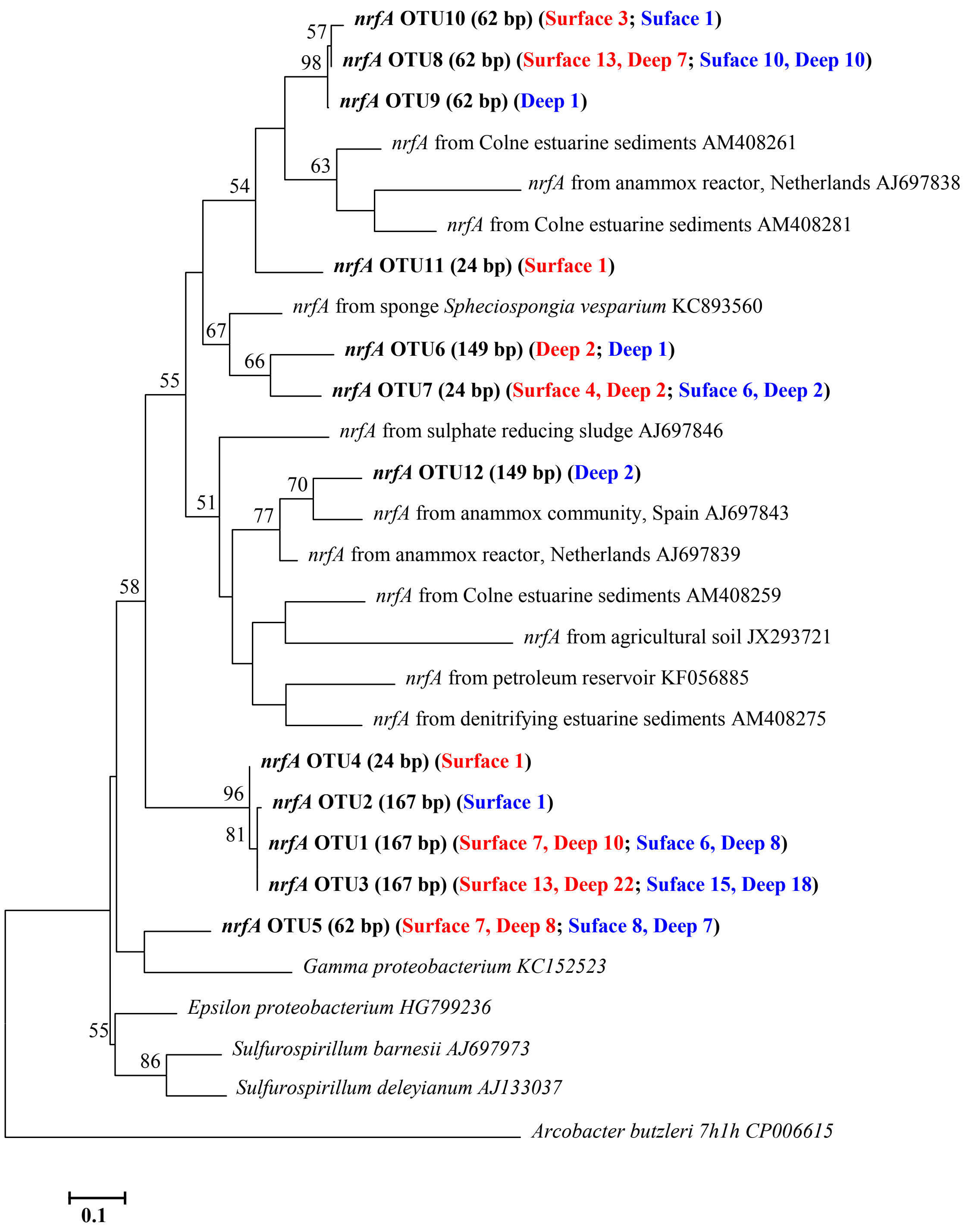


**Supplementary Figure 5** Neighbor-joining phylogenetic tree of DNRA bacteria *nrfA* gene sequences. Bootstrap values greater than 50% of 1000 resamplings are shown near nodes. The scale indicates the number of nucleotide substitutions per site. GenBank accession numbers are shown for sequences from other studies. Numbers in parentheses followed each OTU indicate the number of sequences recovered from surface and (or) deep part of the sediment cores in April (red) and October (blue). OTUs are defined by <2% divergence in nucleotides.

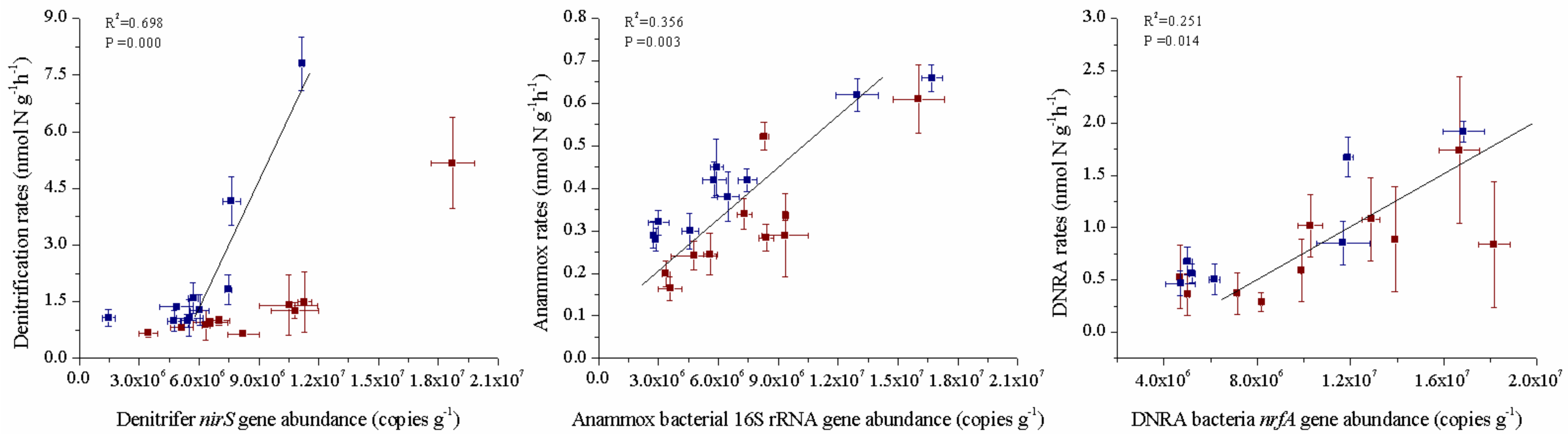


**Supplementary Figure 6** Pearson's correlations between dissimilatory nitrate-reducing rates and associated bacterial gene abundances in intertidal marshes. Blue: April; Wine: October. Error bars indicates s.d. (n = 3).

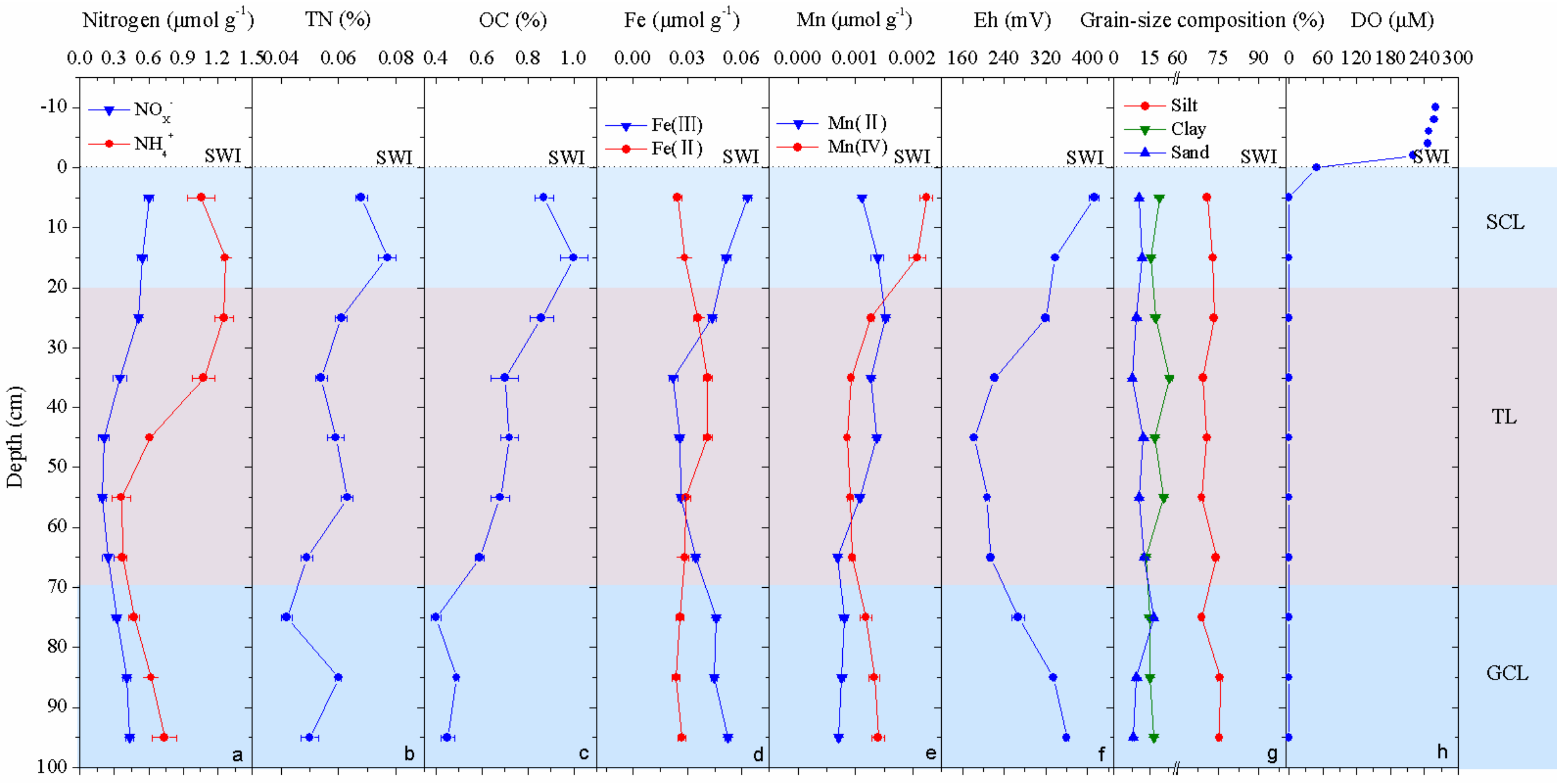

Nitrogen (μmol g-1)
TN (%)
OC (%)
Fe (μmol g-1)
Mn (μmol g-1)
Eh (mV)
Grain-size composition (%)
DO (μM)
Depth (cm)
NOx-
NH4+
Fe(III)
Fe(II)
Mn(II)
Mn(IV)
Silt
Clay
Sand
SWI
SCL
TL
GCL
a
b
c
d
e
f
g
h

**II**

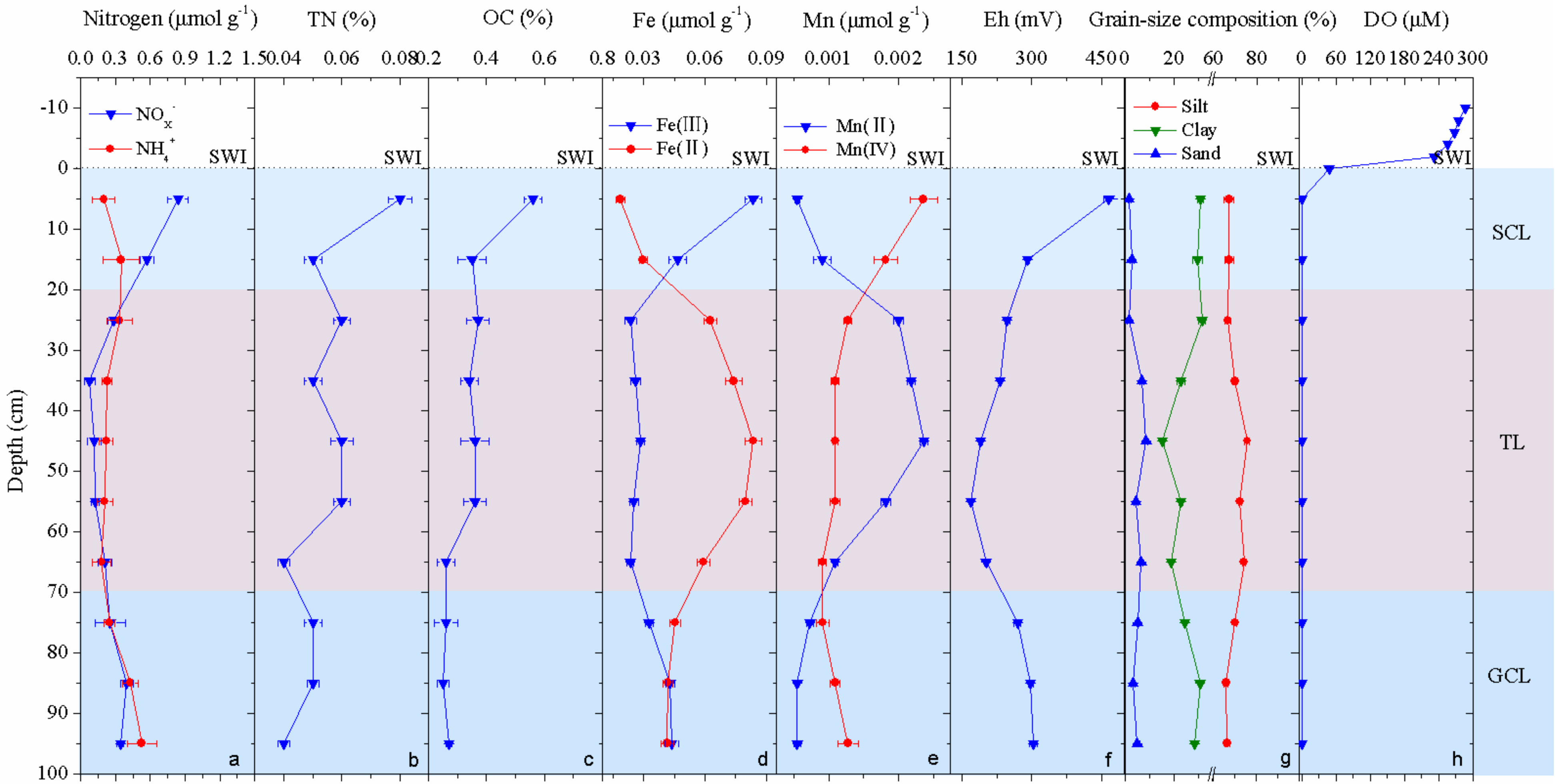


**Supplementary Figure 7** Vertical distributions of $NO_x^-$ ($NO_3^-$ + $NO_2^-$) and $NH_4^+$ (a), total nitrogen (TN, b), organic carbon (OC, c), Fe (d), Mn (e), Eh (f), grain-size composition (g), and oxygen (h) in intertidal marsh sediments in April (**I**) and October (**II**). SCL: Sediment-water interface Controlled Layer; TL: Transition Layer; GCL: Groundwater Controlled Layer. Triplicate samples were analyzed to get mean and standard deviation. Concentrations of $NO_x^-$, $NH_4^+$, total nitrogen, organic carbon, Fe, and Mn are expressed on a dry sediment weight basis.

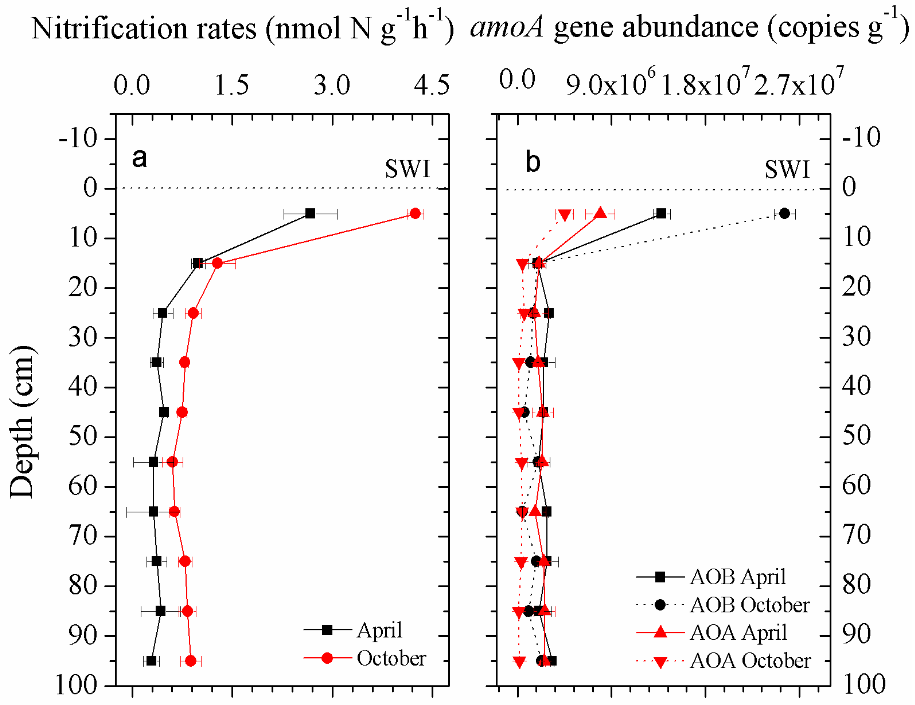


**Supplementary Figure 8** Potential nitrification rates and *amoA* gene abundance in intertidal sediment cores. Error bars indicates s.d. (n = 3).

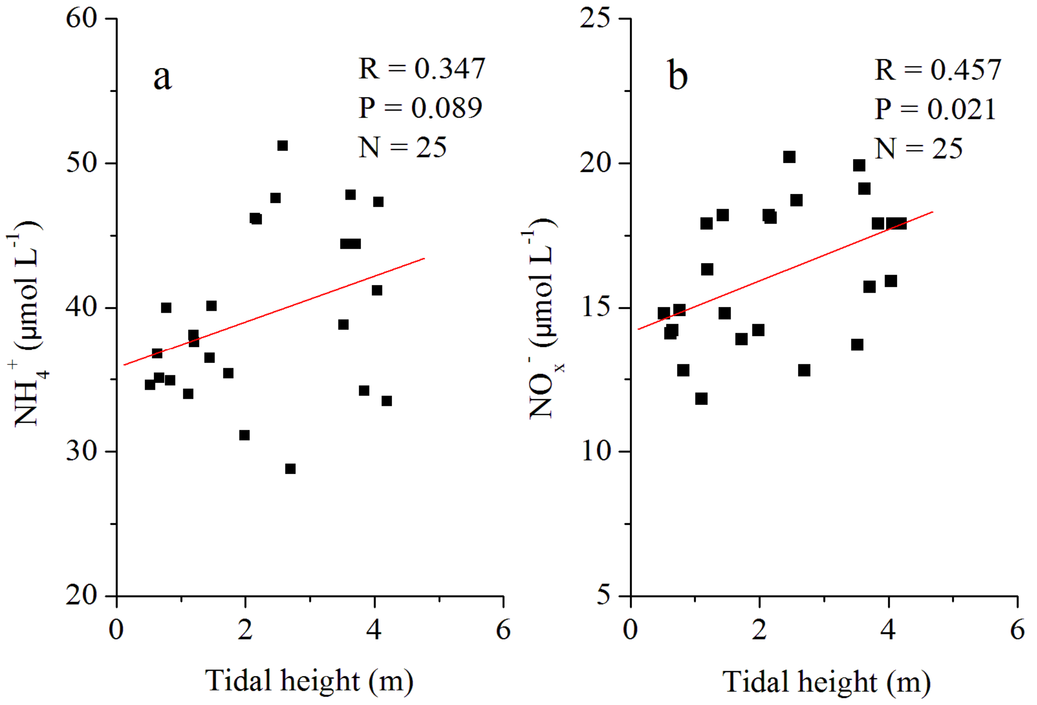


**Supplementary Figure 9** Pearson's correlation analyses of tidal height with dissolved inorganic nitrogen (a: $NH_4^+$; b: $NO_x^-$ = $NO_3^-$ + $NO_2^-$) in 5-cm depth porewater during the two daily tidal cycles. These observed relationships show that the tidal cycle plays an important role in modulating the concentrations of $NH_4^+$ and $NO_x^-$ in surface sediment porewater. Compared with tidal emersion, relatively high concentrations of $NO_x^-$ and $NH_4^+$ concentrations during tidal immersion were perhaps attributed to the transport of $NO_3^-$ from $NO_3^-$-enriched tidal water to sediments, part of which was converted rapidly to $NH_4^+$ via DNRA. Additionally, $NH_4^+$ consumption in surface sediments via nitrification is enhanced during tidal emersion compared with tidal immersion, and thus might also contribute to the tidal changes of $NH_4^+$ and $NO_x^-$.

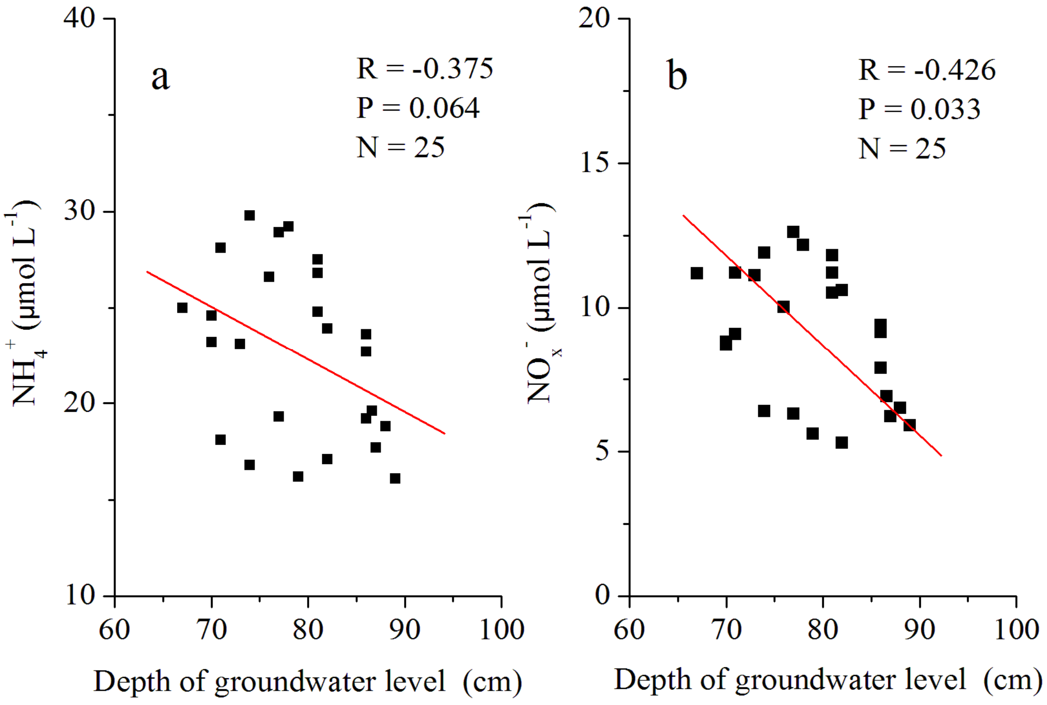


**Supplementary Figure 10** Pearson's correlation analyses of depth of groundwater level with dissolved inorganic nitrogen (a: $NH_4^+$; b: $NO_x^-$ = $NO_3^-$ + $NO_2^-$) in 80-cm depth porewater during the two daily tidal cycles. Shallower groundwater level occurs at high tides while deeper groundwater level occurs at low tides. These observed relationships imply that the fluctuations in the groundwater level play an important role in modulating the concentrations of $NH_4^+$ and $NO_x^-$ in porewater of groundwater controlled layer (GCL). When the groundwater level rose, the increase in $NO_x^-$ and $NH_4^+$ concentrations was perhaps due to recharging by the rising groundwater.

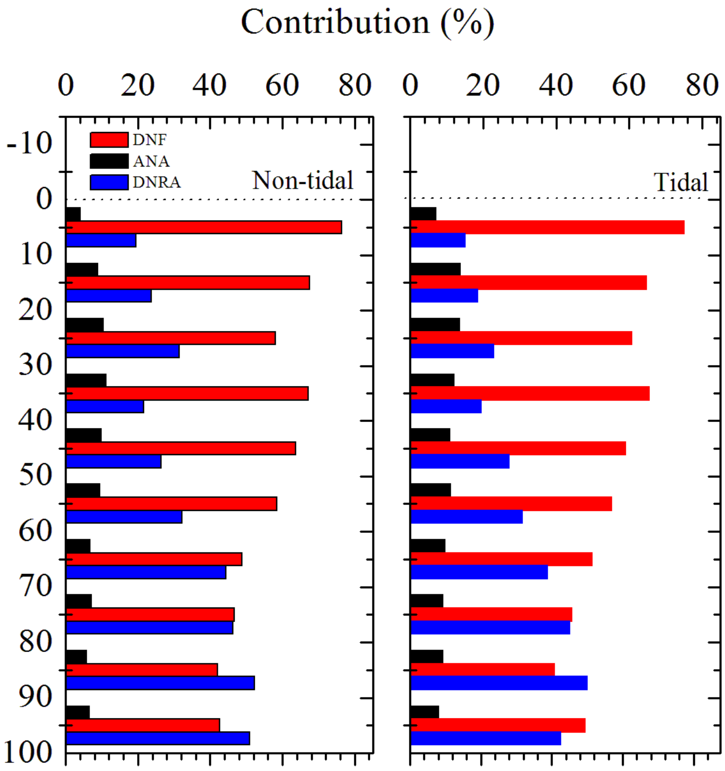


**Supplementary Figure 11** Contributions of denitrification (DNF), anammox (ANA) and DNRA to total nitrate reduction in the tidal simulation experiment. Tidal treatment was incubated in dark for 30 days under periodic immersion and emersion. Non-tidal was the control group without tidal treatment during the 30-day incubation.

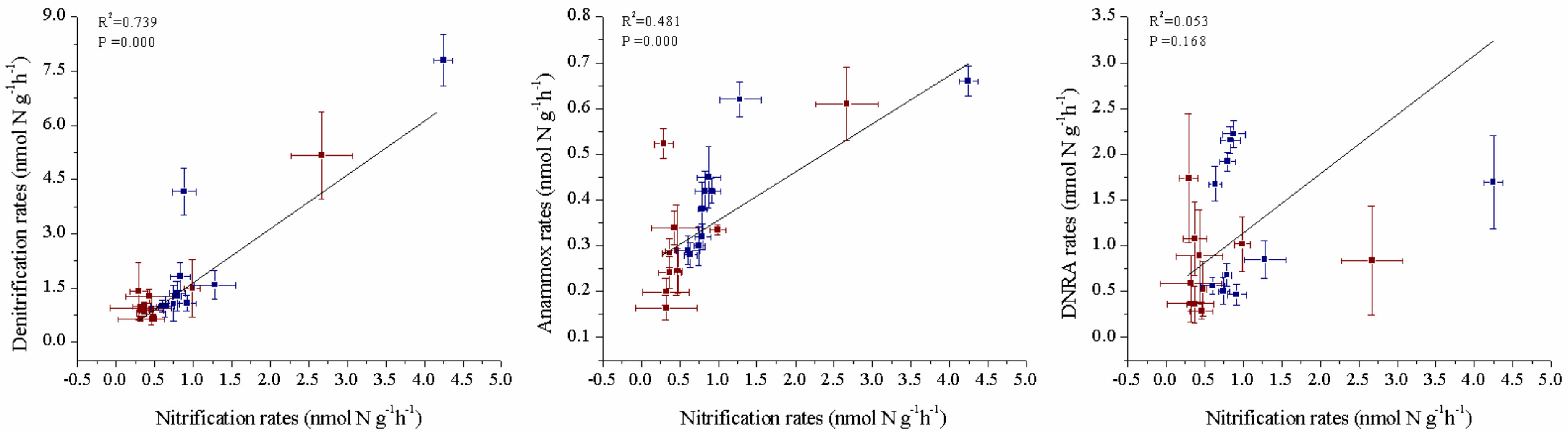


**Supplementary Figure 12** Pearson's correlations between potential nitrification rates and dissimilatory nitrate reduction rates. Blue: April; Wine: October. Error bars indicates s.d. (n = 3).

**Supplementary Table 1** Characteristics of clone libraries in this study.

| Target gene | Season | Sample | No. of clones | OTUs[a] | Chao1[b] | Shannon[c] | 1/Simpson[d] | Coverage (%)[e] |
|---|---|---|---|---|---|---|---|---|
| Denitrifier *nirS* gene | April | Surface | 109 | 22 | 23.5 | 2.57 | 7.95 | 93.6 |
| | | Deep | 101 | 19 | 19.0 | 2.49 | 7.70 | 100 |
| | October | Surface | 110 | 18 | 18.3 | 2.36 | 6.58 | 98.6 |
| | | Deep | 111 | 19 | 19.5 | 2.38 | 6.70 | 97.4 |
| Anammox 16S rRNA gene | April | Surface | 52 | 9 | 9.5 | 1.95 | 6.87 | 94.7 |
| | | Deep | 51 | 9 | 9.0 | 1.82 | 4.60 | 100 |
| | October | Surface | 45 | 5 | 6.0 | 1.16 | 2.87 | 83.3 |
| | | Deep | 45 | 4 | 4.0 | 1.05 | 2.63 | 100 |
| DNRA *nrfA* gene | April | Surface | 49 | 8 | 9.0 | 1.79 | 5.68 | 88.9 |
| | | Deep | 51 | 6 | 6.0 | 1.50 | 3.90 | 100 |
| | Ocobert | Surface | 47 | 7 | 8.0 | 1.68 | 5.20 | 87.5 |
| | | Deep | 49 | 8 | 8.3 | 1.69 | 4.72 | 96.0 |

[a]OTUs are defined at 3% nucleotide acid divergence for *nirS* and anammox 16S rRNA sequences, and at 2% for *nrfA* sequences.
[b]Nonparametric statistical predictions of total richess of OTUs based on distribution of singletons and doubletons.
[c]Shannon diversity index. A higher number represents more diversity.
[d]Reciprocal of Simpson's diversity index. A higher number represents more diversity.
[e]Percentage of coverage: percentage of observed number of OTUs divided by Chao1 estimate.

**Supplementary Table 2** Denitrification, anammox and DNRA rates and their respective contributions to total nitrate loss in this study and other estuarine and tidal ecosystems. NA means no data available.

| Locations | Nitrate reduction rates ($\mu$mol N $m^{-2}$ $h^{-1}$; Italic: nmol N $g^{-1}$ $h^{-1}$) | | | Respective contribution (%) | | | |
|---|---|---|---|---|---|---|---|
| | Denitrification | ANAMMOX | DNRA | Denitrification | ANAMMOX | DNRA | Refs |
| York River marsh | *0-67.4* | *NA* | *0-23.6* | 74.2 | NA | 25.8 | 5 |
| Hythe Estuary | 385 | 157 | 320 | 45 | 18 | 37 | 6 |
| Plum Island Sound | *9-31* | *0-0.2* | *NA* | >97 | <1-3 | NA | 7 |
| Plum Island Sound (marsh) | 5.2 | NA | 3.9 | 57 | NA | 43 | 8 |
| Mae Klong Estuary | 0-7.4 | NA | 0.3-22.8 | 26 | NA | 74 | 9 |
| Copnano bay | 27.7-40.1 | 0.26-1.6 | 1.4-3.8 | 98.03-99.3 | 0.4-1.45 | 0.3-0.52 | 10 |
| Pearl River Estuary | *4.8-19.4* | *0.04-1.4* | *NA* | 93-99.5 | 0.5-7 | NA | 11 |
| Yangtze Estuary | *12.0-68.0* | *0.94-6.61* | *NA* | 87.1-93.4 | 6.6-12.9 | NA | 12 |
| Lugano south basin | 5.9-57.2 | 0-3.8 | 0-6.2 | >82 | <6 | <12 | 13 |
| Colne Estuary | 12.3-415.6 | NA | 106.3-679.1 | 9.3-38.0 | NA | 62.0-90.7 | 14 |
| Seine Estuary | *15.5-21.9* | *0.6-1.3* | *NA* | 92-97 | 3-8 | NA | 15 |
| Chongming, Yangtze Estuary | *0.64-7.80* | *0.16-0.66* | *0.29-2.22* | 33.7-78.1 | 6.5-24.1 | 12.7-56.8 | This study |

**Supplementary Table 3** Primers and PCR protocols used in this study.

| Target gene | Primers | Sequence (5'-3') | Refs | PCR conditions | |
|---|---|---|---|---|---|
| *Planctomycetales* 16S rRNA gene | Pla46f | GGATTAGGCATGCAAGTC | 16 | 94 °C for 4 min, 30 × [95 °C for 45 s, 59 °C for 50 s, 72 °C for 1 min 40 s], 72 °C for 5 min | PCR[a] |
| | 1390r | GACGGGCGGTGTGTACAA | 17 | | |
| Anammox 16S rRNA gene | Amx368f | TTCGCAATGCCCGAAAGG | 18 | 94 °C for 4 min, 30 × [95 °C for 45 s, 59 °C for 50 s, 72 °C for 1 min], 72 °C for 5 min | PCR[b] |
| | Amx820r | AAAACCCCTCTACTTAGTGCCC | 19 | | |
| Anammox 16S rRNA gene | Amx-808-F | ARCYGTAAACGATGGGCACTAA | 20 | 50 °C for 2 min, 95 °C for 10 min, 45 × [95 °C for 30 s, 55 °C for 30 s, 72 °C for 30 s] | qPCR[c] |
| | Amx-1040-R | CAGCCATGCAACACCTGTRATA | | | |
| Denitrifier *nirS* gene | cd3aF | GTSAACGTSAAGGARACSGG | 21 | 94 °C for 4 min, 30 × [95 °C for 45 s, 55 °C for 45 s, 72 °C for 50 s], 72 °C for 5 min | PCR[b] |
| | R3cd | GASTTCGGRTGSGTCTTGA | 22 | 50 °C for 2 min, 95 °C for 10 min, 40 × [95 °C for 30 s, 57 °C for 45 s, 72 °C for 50 s] | qPCR[c] |
| DNRA *NrfA* gene | NrfAF2aw | CARTGYCAYGTBGARTA | 23 | 94 °C for 4 min, 30 × [95 °C for 45 s, 53 °C for 45 s, 72 °C for 30 s], 72 °C for 5 min | PCR[b] |
| | NrfAR1 | TWNGGCATRTGRCARTC | 24 | 50 °C for 2 min, 95 °C for 10 min, 45 × [95 °C for 30 s, 55 °C for 40 s, 72 °C for 30 s] | qPCR[c] |
| Bacterial *amoA* gene | amoA-1F | GGGGTTTCTACTGGTGGT | 3 | 50 °C for 2 min, 95 °C for 10 min, 45 × [95 °C for 30 s, 58 °C for 40 s, 72 °C for 1 min] | qPCR[c] |
| | amoA-2R | CCCCTCKGSAAAGCCTTCTTC | | | |
| Archaeal *amoA* gene | Arch-amoAF | STAATGGTCTGGCTTAGACG | 4 | 50 °C for 2 min, 95 °C for 10 min, 45 × [95 °C for 30 s, 56 °C for 45 s, 72 °C for 1 min] | qPCR[c] |
| | Arch-amoAR | GCGGCCATCCATCTGTATGT | | | |

[a]PCR was performed in a total volume of 25 μL containing 10 × PCR buffer (without $MgCl_2$, Sangon, China) 2.5 μL, $MgCl_2$ (25 mM, Sangon) 2 μL, dNTP (each 10 mM, Sangon) 0.5 μL, each primer (10 μM, Sangon) 0.5 μL, Taq DNA Polymerase (5 U $\mu L^{-1}$, Sangon) 0.5 μL, and template DNA 1 μL.
[b]PCR was performed in a total volume of 50 μL containing 10 × PCR buffer (without $MgCl_2$, Sangon, China) 5 μL, $MgCl_2$ (25 mM, Sangon) 4 μL, dNTP (each 10 mM, Sangon) 1 μL, each primer (10 μM, Sangon) 1 μL, Taq DNA Polymerase (5 U $\mu L^{-1}$, Sangon) 1 μL, and template 1 μL.
[c]The 25 μL qPCR mixture contained 12.5 μL of Maxima SYBR Green/Rox qPCR Master Mix (Fermentas, Lithuania), 1 μL of each primer (10 μM) and 1 μL template DNA.

**Supplementary Table 4** qPCR calibration curve parameters in this study.

| Target gene | Amplicon length (bp) | Slope | y-Intercept | Amplification efficiency (%) | $R^2$ |
|---|---|---|---|---|---|
| Anammox 16S rRNA gene | 275 | -3.434 | 38.33 | 95.5 | 0.9991 |
| Denitrifier *nirS* gene | 410 | -3.393 | 37.67 | 97.1 | 0.9959 |
| DNRA *nrfA* gene | 254 | -3.459 | 38.45 | 94.6 | 0.9992 |
| Bacterial *amoA* gene | 491 | -3.555 | 38.89 | 91.1 | 0.9964 |
| Archaeal *amoA* gene | 635 | -3.521 | 36.72 | 92.3 | 0.9996 |

## Supplementary References:


1. Bernhard, A. E., Tucker, J., Giblin, A. E. & Stahl, D. A. Functionally distinct communities of ammonia-oxidizing bacteria along an estuarine salinity gradient. *Environ. Microbiol.* **9,** 1439-1447 (2007).
2. Zheng, Y. L. *et al*. Community dynamics and activity of ammonia-oxidizing prokaryotes in intertidal sediments of the Yangtze Estuary. *Appl. Environ. Microb.* **80,** 408-419 (2014).
3. Rotthauwe, J. H., Witzel, K. P. & Liesack, W. The ammonia monooxygenase structural gene *amoA* as a functional marker: molecular finescale analysis of natural ammonia-oxidizing populations. *Appl. Environ. Microbiol.* **63,** 4704-4712 (1997).
4. Francis, C. A., Roberts, K. J., Beman, J. M., Santoro, A. E. & Oakley, B. B. Ubiquity and diversity of ammonia-oxidizing archaea in water columns and sediments of the ocean. *Proc. Natl. Acad. Sci. USA* **102,** 14683-14688 (2005).
5. Tobias, C. R., Anderson, I. C., Canuel, E. A. & Macko, S. A. Nitrogen cycling through a fringing marsh-aquifer ecotone. *Mar. Ecol. Prog. Ser.* **210,** 25-39 (2001).
6. Dong, L. F., Smith, C. J., Papaspyrou, S., Osborn, A. M. & Nedwell, D. B. Changes in benthic denitrification, nitrate ammonification, and anammox process rates and nitrate and nitrite reductase gene abundances along an estuarine nutrient gradient (the Colne Estuary, United Kingdom). *Appl. Environ. Microb.* **75,** 3171-3179 (2009).
7. Koop-Jakobsen, K. & Giblin, A. E. Anammox in tidal marsh sediments: the role of salinity, nitrogen loading, and marsh vegetation. *Estuar. Coast.* **32,** 238-245 (2009).
8. Koop-Jakobsen, K. & Giblin, A. E. The effect of increased nitrate loading on nitrate reduction via denitrification and DNRA in salt marsh sediments, *Limnol. Oceanogr.* **55,** 789-802 (2010).
9. Dong, L. F. *et al*. Dissimilatory reduction of nitrate to ammonium, not denitrification or anammox, dominates benthic nitrate reduction in tropical estuaries. *Lmnol. Oceanogr.* **56,** 279-291 (2011).
10. Hou, L. J., Liu, M., Carini, S. A. & Gardner, W. S. Transformation and fate of nitrate near the sediment-water interface of Copano Bay. *Cont. Shelf. Res.* **35**, 86-94 (2012).
11. Wang, S., Zhu, G. B., Peng, Y. Z., Jetten, M. S. M. & Yin, C. Anammox bacterial abundance, activity, and contribution in riparian sediments of the Pear River Estuary. *Environ. Sci. Technol.* **46,** 8834-8842 (2012).
12. Hou, L. J. *et al*. Anaerobic ammonium oxidation (anammox) bacterial diversity, abundance, and activity in marsh sediments of the Yangtze Estuary. *J. Geophys. Res. Biogeosci.* **118,** 1237-1246 (2013).
13. Wenk, C. B. *et al.* Partitioning between benthic and pelagic nitrate reduction in the Lake Lugano south basin. *Limnol. Oceanogr.* **59,** 1421-1433 (2014).
14. Smith, C. J. *et al*. Seasonal variation in denitrification and dissimilatory nitrate reduction to ammonia process rates and corresponding key functional genes along an estuarine nitrate gradient. *Front. Microbiol.* **6,** 542 (2015).

15. Naeher , S. *et al*. Molecular and geochemical constraints on anaerobic ammonium oxidation (anammox) in a riparian zone of the Seine Estuary (France). *Biogeochemistry* **123,** 237-250 (2015).

16. Neef, A., Amann, R. I., Schlesner, H. & Schleifer, K. H. Monitoring a widespread bacterial group: *in situ* detection of *Planctomycetes* with 16S rRNA-targeted probes. *Microbiology* **144,** 3257-3266 (1998).

17. Zheng, D., Alm, E.W., Stahl, D. A. & Raskin, L. Characterization of universal small-subunit rRNA hybridization probes for quantitative molecular microbial ecology studies. *Appl. Environ. Microbiol.* **62,** 4504-4513 (1996).

18. Schmid, M. C. *et al*. Candidatus '*Scalindua brodae*', sp. nov., Candidatus '*Scalindua wagneri*', sp. nov., two new species of anaerobic ammonium oxidizing bacteria. *Syst. Appl. Microbiol.* **26,** 529-538 (2003).

19. Schmid, M. C. *et al.* Molecular evidence for genus level diversity of bacteria capable of catalyzing anaerobic ammonium oxidation. *Syst. Appl. Microbiol.* **23,** 93-106 (2000).

20. Hamersley, M. R. *et al*. (2007). Anaerobic ammonium oxidation in the Peruvian oxygen minimum zone. *Limnol. Oceanogr.* **52,** 923-933.

21. Michotey, V., Mejean, V. & Bonin, P. Comparison of methods for quantification of cytochrome *cd1*-denitrifying bacteria in environmental marine samples. *Appl. Environ. Microbiol.* **66,** 1564-1571 (2000).

22. Throbäck, I. N., Enwall, K., Jarvis, A. & Hallin, S. Reassessing PCR primers targeting *nirS*, *nirK* and *nosZ* genes for community surveys of denitrifying bacteria with DGGE. *FEMS Microbiol. Ecol.* **49,** 401-417 (2004).

23. Welsh, A. *et al.* Refined NrfA Phylogeny Improves PCR-Based *nrfA* Gene Detection. *Appl. Environ. Microbiol.* **80,** 2110-2119 (2014).

24. Mohan, S. B., Schmid, M., Jetten, M. & Cole, J. Detection and widespread distribution of the *nrfA* gene encoding nitrite reduction to ammonia, a short circuit in the biological nitrogen cycle that competes with denitrification. *FEMS Microbiol. Ecol.* **49,** 433-443 (2004).
